# Evidence that a neutrophil–keratinocyte crosstalk is an early target of IL-17A inhibition in psoriasis

**DOI:** 10.1111/exd.12710

**Published:** 2015-05-08

**Authors:** Kristian Reich, Kim A Papp, Robert T Matheson, John H Tu, Robert Bissonnette, Marc Bourcier, David Gratton, Rodion A Kunynetz, Yves Poulin, Les A Rosoph, Georg Stingl, Wolfgang M Bauer, Janeen M Salter, Thomas M Falk, Norbert A Blödorn-Schlicht, Wolfgang Hueber, Ulrike Sommer, Martin M Schumacher, Thomas Peters, Ernst Kriehuber, David M Lee, Grazyna A Wieczorek, Frank Kolbinger, Conrad C Bleul

**Affiliations:** 1 Dermatologikum Hamburg and SCIderm Research InstituteHamburg, Germany; 2 Probity Medical Research IncWaterloo, ON, Canada; 3 Oregon Medical Research Center PCPortland, OR, USA; 4 Skin Search of RochesterRochester, NY, USA; 5 Innovaderm Research IncMontreal, QC, Canada; 6 Dermatology ClinicMoncton, NB, Canada; 7 International Dermatology ResearchMontreal, QC, Canada; 8 Ultranova SkincareBarrie, ON, Canada; 9 Centre de Recherche Dermatologique du Québec MétropolitainQuebec City, QC, Canada; 10 North Bay Dermatology CentreNorth Bay, ON, Canada; 11 Division of Immunology, Allergy and Infectious Diseases, Department of Dermatology, Medical University of ViennaVienna, Austria; 12 Novartis Institutes for BioMedical ResearchBasel, Switzerland

**Keywords:** IL-17A, neutrophils, psoriasis, secukinumab

## Abstract

The response of psoriasis to antibodies targeting the interleukin (IL)-23/IL-17A pathway suggests a prominent role of T-helper type-17 (Th17) cells in this disease. We examined the clinical and immunological response patterns of 100 subjects with moderate-to-severe psoriasis receiving 3 different intravenous dosing regimens of the anti-IL-17A antibody secukinumab (1 × 3 mg/kg or 1 × 10 mg/kg on Day 1, or 3 × 10 mg/kg on Days 1, 15 and 29) or placebo in a phase 2 trial. Baseline biopsies revealed typical features of active psoriasis, including epidermal accumulation of neutrophils and formation of microabscesses in >60% of cases. Neutrophils were the numerically largest fraction of infiltrating cells containing IL-17 and may store the cytokine preformed, as IL-17A mRNA was not detectable in neutrophils isolated from active plaques. Significant clinical responses to secukinumab were observed 2 weeks after a single infusion, associated with extensive clearance of cutaneous neutrophils parallel to the normalization of keratinocyte abnormalities and reduction of IL-17-inducible neutrophil chemoattractants (e.g. *CXCL1*, *CXCL8*); effects on numbers of T cells and CD11c-positive dendritic cells were more delayed. Histological and immunological improvements were generally dose dependent and not observed in the placebo group. In the lowest-dose group, a recurrence of neutrophils was seen in some subjects at Week 12; these subjects relapsed faster than those without microabscesses. Our findings are indicative of a neutrophil–keratinocyte axis in psoriasis that may involve neutrophil-derived IL-17 and is an early target of IL-17A-directed therapies such as secukinumab.

## Introduction

Several key mechanisms have been proposed as initiating and maintaining psoriasis, including the activation of dendritic cells by complexes of self-DNA and the antimicrobial peptide cathelicidin LL-37, the presentation of putative auto-antigens to T lymphocytes, and the release of pro-inflammatory mediators such as interleukin (IL)-23, tumor necrosis factor-*α* (TNF-*α*) and IL-17A, leading to the activation of keratinocytes, which close the loop by producing antimicrobial peptides such as LL-37, chemoattractants and *β*-defensins 1–3. A central element in current disease models is the T-helper type-17 cell subset (Th17). Consistent with this concept, IL-23 (believed to mediate the crosstalk between dendritic and Th17 cells) and IL-17A (a key effector cytokine released from Th17 cells) have emerged as attractive targets for the development of new therapies for psoriasis. The clinical efficacy of ustekinumab – an anti-p40 monoclonal antibody that blocks IL-12 and IL-23 – and antibodies directed against IL-17A and the IL-17 receptor A chain has been taken as evidence that the IL-23/Th17 axis is indeed central to the pathophysiological cascade of psoriasis 4–9. More recently, inflammatory cells other than Th17 – in particular, neutrophils, a numerically important component of the infiltrate in active psoriasis – have also been suggested as sources of IL-17A 10,11. IL-17A not only contributes to the epidermal abnormalities typical of psoriasis, but also induces the expression of chemokines in keratinocytes such as GRO-*α* (*CXCL1*) and IL-8 (*CXCL8*), which orchestrate the recruitment of neutrophils to psoriatic lesions 12.

To further dissect the role of different sources of IL-17A in psoriasis, this study investigated serial biopsies taken from active plaques at Baseline and treatment Weeks 2 and 12 during a phase 2 trial in which subjects with moderate-to-severe psoriasis received 1 of 3 induction regimens with the IL-17A-selective antibody secukinumab. Two weeks after a single intravenous infusion of secukinumab, IL-17-containing neutrophils had almost completely disappeared parallel to a striking normalization of psoriatic epidermal changes and reduction of keratinocyte-derived, IL-17-inducible neutrophil chemoattractants. Our findings suggest that IL-17A is not only part of an adaptive immune circuit involving specific T-cell subsets, but also part of an innate axis between neutrophils and keratinocytes that serves as an early target of anti-IL-17A antibodies.

## Materials and methods

### Subjects and study design

A total of 130 subjects were enrolled at 14 sites in the United States and Canada between December 2008 and July 2009. Subjects aged 18–65 years were eligible if they had chronic plaque psoriasis for ≥6 months, coverage of ≥10% of their body surface area with plaques, an Investigator’s Global Assessment score ≥3 (scale of 0–5) and a Psoriasis Area and Severity Index (PASI) score ≥12. The study consisted of a 12-week induction period and a follow-up period up to Week 56 after the first dose of study drug. Eligible subjects were randomly assigned to receive 1 of 3 regimens of intravenous secukinumab (AIN457; Novartis Pharmaceuticals, Basel, Switzerland), a fully human immunoglobulin-G1*κ* monoclonal antibody selective for IL-17A, or placebo in a 3:3:3:1 ratio (details regarding sample size calculation, randomization and blinding are provided in the Supporting Information). There were low- and mid- single-dose cohorts who received secukinumab 3 and 10 mg/kg, respectively, infused on Day 1 (with placebo administered on Day 15 and Day 29) and a high-dose cohort who received three infusions of secukinumab 10 mg/kg at 2-week intervals. Infusions were given over 2 h. The primary objectives were to compare the change from Baseline in PASI score at Week 12 between cohorts and to determine the proportions of subjects who did not relapse at any time through Week 56.

Secondary efficacy endpoints included the proportions of subjects with ≥50%, ≥75% and ≥90% improvements from Baseline in PASI (PASI50/PASI75/PASI90), and changes in Investigator’s Global Assessment and Dermatology Life Quality Index scores. One study site with 30 subjects was terminated prematurely because of data-quality concerns; the efficacy and safety data for 100 subjects (excluding those from the terminated site) are presented in this analysis. The study was conducted according to the Declaration of Helsinki. The study protocol and all amendments were approved by the central independent ethics committees or institutional review boards in the participating countries. All study subjects provided written informed consent for their participation. The full study protocol is available from the sponsor (ClinicalTrials.gov identifier NCT00805480; date of registration: 5 December 2008).

### RNA extraction, NanoString nCounter® and quantitative reverse-transcription–polymerase chain reaction gene expression analysis of skin biopsies

Four-millimetre punch biopsies were obtained from a representative psoriatic plaque at Baseline and from the same plaque at Weeks 2 and 12. All 100 subjects included here had Baseline biopsies taken and biopsies from Weeks 2 and 12 were available from almost all subjects for analysis (Fig. S1). One part of each biopsy was immediately embedded in optimal cutting temperature compound (Tissue-Tek® O.C.T.™ Compound, Sakura Finetek, Alphen aan den Rijn, the Netherlands), stored at −70°C and later processed for RNA extraction, while the other part was fixed in paraformaldehyde and used for histology and immunohistochemistry. All biopsies were handled and analysed by personnel blinded to treatment and time points. RNA was isolated using the RNeasy Fibrous Tissue Mini Kit (Qiagen NV, Venlo, the Netherlands) as described in the Supporting Information.

To analyse a broader set of mRNAs with high sensitivity, a subset of samples was processed with the nCounter Prep Station and Digital Analyzer and tested with a custom-designed nCounter Gene Expression CodeSet Maestro (NanoString Technologies, Seattle, WA, USA) containing probes for 180 psoriasis-related transcripts, nine candidate reference transcripts for normalization and two gender control transcripts. Probe sequences for genes reported in this study are shown in Table S1; further details of the methodology and the control quantitative reverse-transcription-polymerase chain reaction performed for *IL17A* and *IFNG* are given in the Supporting Information.

### Immunohistochemistry and immunofluorescence

Epidermal thickness and parakeratosis, as well as staining of Ki67, CD11c, CD3, IL-17, myeloperoxidase, and mast cell tryptase, were evaluated on paraffin-embedded, haematoxylin/eosin-stained sections, alone or in combination with immunohistochemistry using a prospectively defined semi-quantitative scoring system on digitally scanned images (AxioVision SE64 Rel. 4.8; Carl Zeiss Microscopy, Oberkochen, Germany; Fig. S2). Results were confirmed by automated digital imaging of selected sections. Immunohistochemical stainings were performed according to the manufacturer’s instructions, using the Dako REAL™ Detection System, alkaline phosphatase/RED, rabbit/mouse (Dako, Glostrup, Denmark) in an automated staining system (Dako Autostainer Plus, Dako). Double immunofluorescence stainings of IL-17 vs tryptase and myeloperoxidase, respectively, were performed manually. Slides were mounted with ProLong® Gold Antifade Mountant with DAPI (Life Technologies, Grand Island, NY, USA). Image acquisition was performed on an LSM 700 confocal microscope (Carl Zeiss Microscopy). Lists of antibodies and procedural details are provided in the Supporting Information.

### Analysis of peripheral blood T cells and isolated peripheral blood and skin leucocyte subsets

Surface markers on peripheral T-lymphocyte subsets and the stimulated expression of selected cytokines were assessed by flow cytometry. Percentages of Th17, Th1 and regulatory T cells (Tregs) were determined as described in the Supporting Information. Data acquisition was performed on a BD FACSCanto™ II (Becton, Dickinson and Company, Franklin Lakes, NJ, USA). Leucocyte subsets were also isolated from peripheral blood and psoriatic skin samples and analysed by quantitative polymerase chain reaction as described in the Supporting Information.

### Statistics

Efficacy and pharmacodynamic parameters were evaluated in all subjects who received ≥1 dose of study medication and had no major protocol deviations that could affect these parameters; safety was evaluated in all subjects who received ≥1 dose of study medication. The change from Baseline in PASI score in each secukinumab group compared with the placebo group was evaluated using the Wilcoxon rank-sum test. The proportions of subjects who did not relapse in the secukinumab 1 × 10 and 3 × 10 mg/kg groups compared with the 1 × 3 mg/kg group were evaluated using Fisher’s exact test. Subjects lost to follow-up were considered relapsed on the day of the first visit without available PASI data. The relapse time of subjects who did not relapse during the complete course of the clinical trial was set to 56 weeks (end of study). The significance of the difference between these subject groups was assessed using a 2-sided Wilcoxon rank-sum test. *P-*values corrected for ties are reported. NanoString data were log_2_-transformed. For assessment of the dose and time effect on the investigated transcripts, means of the log_2_-transformed counts and standard errors of the means were plotted.

## Results

During a 12-week induction period, subjects received either one infusion with secukinumab 3 or 10 mg/kg, respectively, on Day 1 or 3 infusions of secukinumab 10 mg/kg on Days 1, 15 and 29 or respective placebo infusions and were followed up to Week 56 as described. Subjects across all groups had a mean Baseline PASI of approximately 19. Details of the study design and the Baseline characteristics of included subjects are shown in Fig. S3 and Table S2, respectively. By Week 12 and by Week 56, 9 (9%) and 38 (38%) of the subjects had discontinued from the trial, respectively (Fig. S1).

### Efficacy

**Figure 1:**
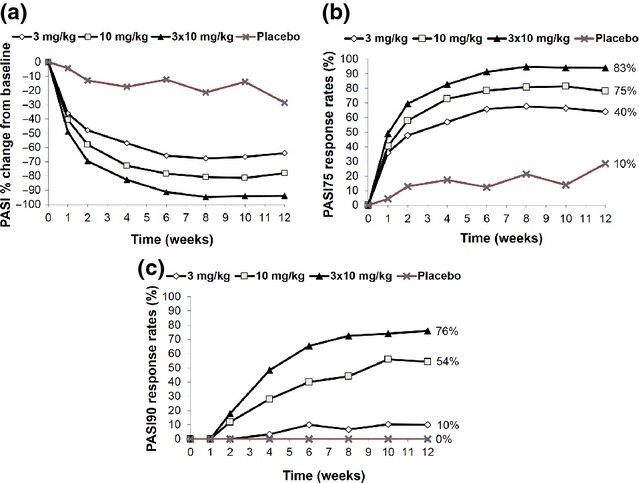
Psoriasis Area and Severity Index (PASI) response rates over time in subjects receiving secukinumab or placebo. (a) % change in PASI from Baseline. (b) ≥75% reduction from Baseline in PASI (PASI75). (c) ≥90% reduction from Baseline in PASI (PASI90).

All 3 secukinumab dose regimens resulted in statistically significant improvements in mean PASI scores compared with placebo at Week 12 (Fig.1a). The PASI responses were rapid, being detectable by 1–2 weeks after dosing and reaching maximum improvement by 6–8 weeks. Consistently, large percentages of subjects achieved PASI responses at Week 12 in each of the active treatment groups, with the highest percentage of responders in the secukinumab 3 × 10 mg/kg group (82.6% and 75.9% for PASI75 and PASI90, respectively), followed by the 1 × 10 mg/kg group (75.0% and 54.2%) and the 1 × 3 mg/kg group (40% and 10%; Fig.1b, c). More than 80% of subjects in all secukinumab groups showed improved Investigator’s Global Assessment scores by Week 2, with these improvements continuing through Week 12, and the mean Dermatology Life Quality Index decreased in all 3 secukinumab groups vs the placebo group (data not shown).

Median times to relapse (defined as the time from the first day of dosing to the day when ≥50% of the maximal PASI response was lost) were 24.2 weeks in the secukinumab 1 × 3 mg/kg group, 28.4 weeks in the 1 × 10 mg/kg group and 40.1 weeks in the 3 × 10 mg/kg group. In the 3 × 10 mg/kg group, about half of the subjects maintained their treatment response through Week 40 (58.6%, 48.3% and 27.8% relapse free at Weeks 36, 40 and 56, respectively).

### Safety

The safety findings during this 56-week trial were consistent with previous observations for secukinumab in similar populations 8,9. The rates of subjects reporting adverse events (AEs) were higher in the secukinumab treatment groups (66.7%, 86.2% and 83.9% with 1 × 3, 1 × 10 and 3 × 10 mg/kg, respectively) than in the placebo group (30.0%). Ten serious AEs were reported in seven subjects. Serious AEs included tibia fracture, fibula fracture, pancreatitis (four AEs in two subjects), myocardial infarction, worsening of psoriasis, angina and worsening of coronary artery disease. In this study, all serious AEs occurred in subjects on secukinumab, and none were considered related to study drug by the investigator. One serious AE (pancreatitis, 3 × 10 mg/kg cohort) resulted in discontinuation of study drug. One case of mild oral candidiasis was reported with the 3 × 10 mg/kg dose. There were no serious infectious AEs, no cases of neutropenia and no significant effects on peripheral T helper–lymphocyte subsets (Th1, Th17), except for a small increase in CD25^high^/CD127^low^/Foxp3^+^ regulatory T cells in the highest-dose group at Week 12 (4.9% at screening vs 5.8% at Week 12; *P *=* *0.0002) (Fig. S4).

### Impact of anti-IL-17A treatment on epidermal and inflammatory changes in psoriatic lesions

**Figure 2:**
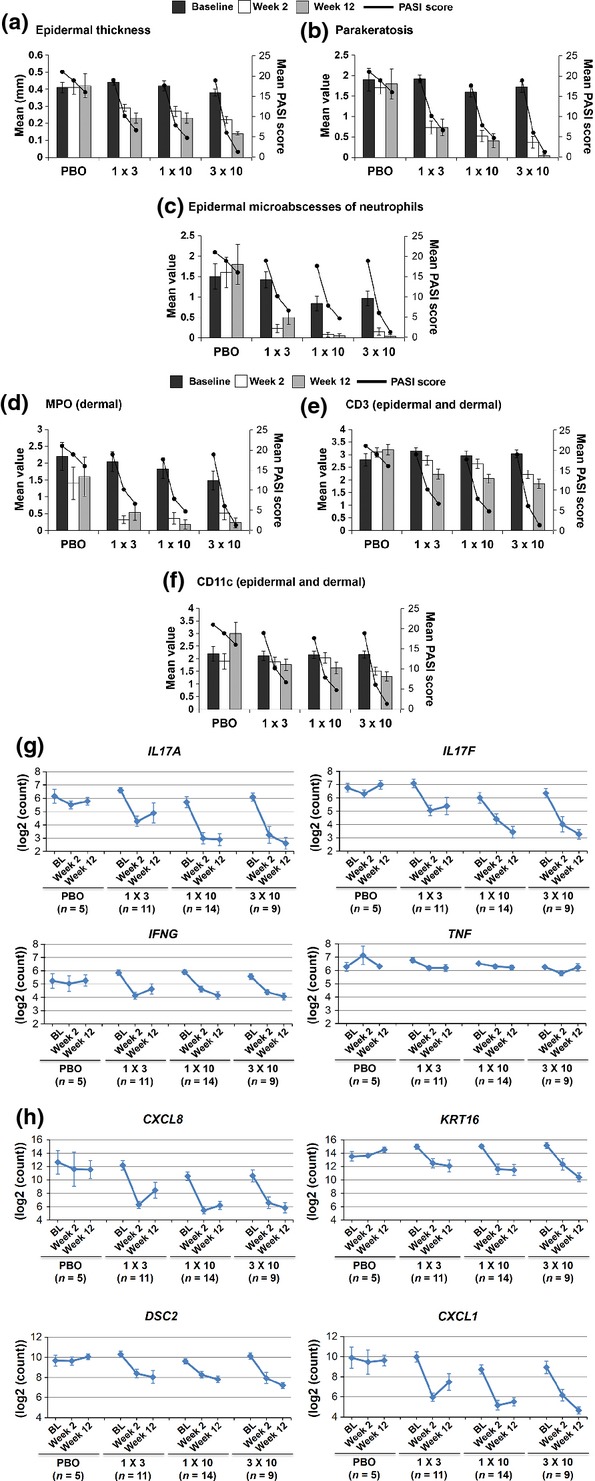
Changes in epidermal and inflammatory parameters in skin lesions during treatment with secukinumab or placebo (a–f; data from all patients). (a–f) Mean values for (a) epidermal thickness in mm (normal 0.1–0.2 mm), (b) parakeratosis, (c) epidermal microabscesses of neutrophils, (d) numbers of myeloperoxidase (MPO)-positive neutrophils, (e) CD3-positive T cells and (f) CD11c-positive dendritic cells. Means for (b–f) derived from semi-quantitative scoring system [see the Materials and methods section and Fig. S2; normal = 0, except for CD3 and CD11c (0–1)]. Solid lines indicate mean changes in Psoriasis Area and Severity Index (PASI). (g, h) Mean log_2_-transformed NanoString counts of expression levels of (g) inflammatory cytokines and (h) epidermal markers in the same biopsies. BL, Baseline; PBO, placebo; all graph titles in g) and h) indicate genes, e.g. *CXCL8*, *DSC2*, *IFNG*, *KRT16*, *TNF* indicate genes encoding *IL-8*, desmocollin-2, IFN-*γ*, keratin-16, TNF-*α*. Error bars are ± standard error of the mean.

**Figure 3:**
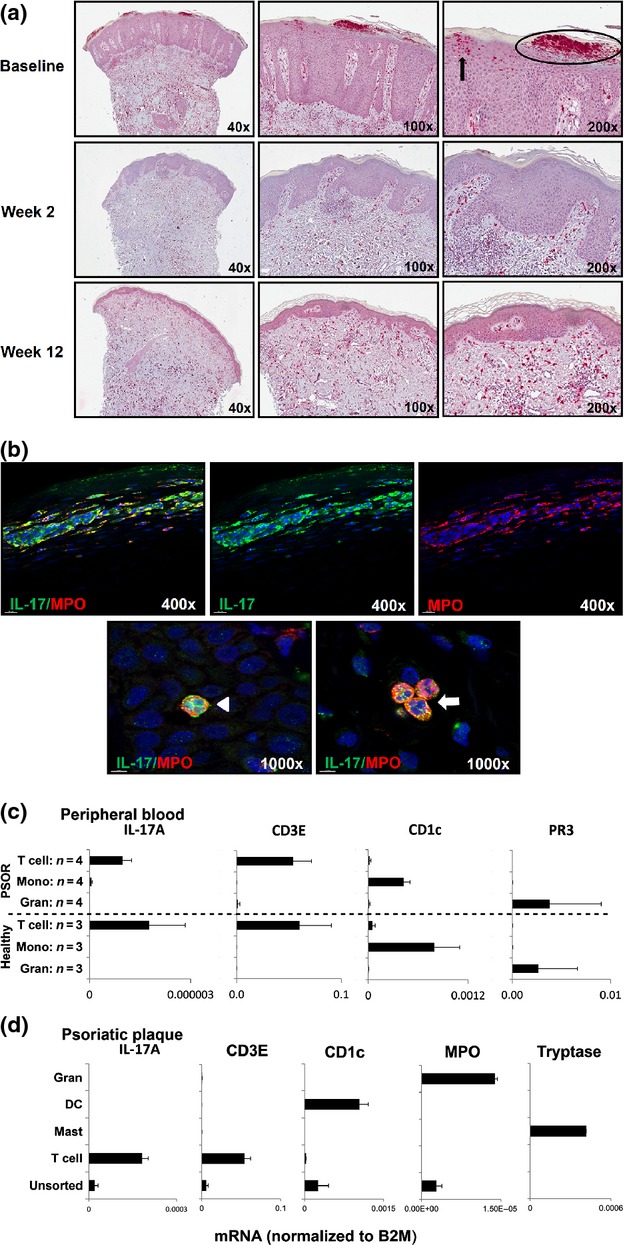
Representative immunohistochemistry of skin biopsies from psoriatic lesions and analysis of *IL17A* expression in peripheral blood and skin leucocyte subsets. (a) Baseline evaluation of IL-17 in a subject from the secukinumab 1 × 10 mg/kg cohort shows prominent staining in epidermal Munro’s microabscesses (oval outline) and spongiform pustules of Kogoj (arrow, top row). Epidermal IL-17 staining was cleared at Week 2, but still evident in dermal T cells and mast cells at Week 12 (middle and bottom rows). (Control sections are shown in Fig. S5.) (b) Immunofluorescence double labelling for IL-17 (green) and myeloperoxidase (MPO; red) with DAPI counterstain confirming IL-17 staining of neutrophils in Munro’s microabscesses (top row), spongiform pustules of Kogoj (arrowhead, bottom left) and neutrophils in dermal vessels (arrow, bottom right). (c) Levels of IL-17A, CD3E, CD1c and proteinase-3 (PR3; to identify neutrophils) mRNA in peripheral blood cells of subjects with psoriasis (*n *=* *4, top row) and healthy donors (*n *=* *3, bottom row). IL-17A mRNA was not detected in more than 95% pure granulocyte fractions (Gran), but observed at low levels in T lymphocytes. Quantification cycle (Cq) for β2-microglobulin (B2M) between 19 and 24 for all samples shown; not detectable: Cq >45. (d) Levels of IL-17A, CD3E, CD1c, MPO (to identify cutaneous neutrophils), and tryptase (to identify cutaneous mast cells) mRNA in cells isolated from psoriatic plaques. One representative experiment of 4 is shown. Samples were run in duplicate for each probe, and quantification was based on ΔΔCq calculations. Arithmetic means ± standard deviation of different donors (a) and of triplicate determinations of individual samples (b) are shown. DC, dendritic cell; Mono, mononuclear; PSOR, psoriasis.

There was a striking reduction in histological correlates of psoriatic epidermal pathology [i.e. epidermal thickness, parakeratosis (Figs2a, b and 3), acanthosis and numbers of Ki67-positive proliferating epidermal cells (Fig. S6a, b)] as early as Week 2. Similarly, mRNA levels for keratin-16 and desmocollin-2 – two keratinocyte markers upregulated in lesional skin – were already significantly reduced at Week 2 (Fig.2h). By Week 12, all epidermal markers had returned to normal levels, particularly in the high-dose cohort.

The most prominent finding with regard to the lesional inflammatory infiltrate was that epidermal microabscesses and dermal neutrophils identified by myeloperoxidase staining – which were present in 65% and 76% of Baseline biopsies, respectively – had almost entirely cleared by Week 2 (Figs2c, d and 3a). In contrast, numbers of T lymphocytes and CD11c-positive dendritic cells decreased more slowly, and elevated levels of T lymphocytes and dendritic cells persisted at Week 12 (Fig.2e, f). Thus, the kinetics of the effects of secukinumab on neutrophils paralleled the rapid epidermal improvement, in contrast to the more steady improvement observed for T lymphocytes and dendritic cells.

Immunohistochemistry and double immunofluorescence labelling for IL-17 and myeloperoxidase with a widely used affinity-purified polyclonal IL-17 antibody 10,11,13 revealed that epidermal neutrophils were the numerically predominant cell type containing IL-17 in active plaques. Blocking experiments with recombinant human IL-17A and IL-17F confirmed that the anti-IL-17 polyclonal antibody primarily detects IL-17A, with some possible cross-reactivity with IL-17F (see Supporting Information and Fig. S5). IL-17 was detected in Munro’s microabscesses (accumulations of neutrophils in the stratum corneum) and spongiform pustules of Kogoj (accumulations of neutrophils in the stratum spinosum), as well as in single neutrophils in the dermis (Fig.3b). Small subsets of lesional T cells (CD3 positive) and dermal mast cells (tryptase positive; Fig. S5) were also found to contain IL-17, in line with earlier findings 10,11. Unlike neutrophils, however, mast cells remained essentially unchanged in number in response to IL-17A blockade (Fig.3a, S6c).

IL-17A mRNA could be detected in T cells, but not in mast cells or neutrophils by quantitative real-time polymerase chain reaction analysis of sorted cell preparations from psoriatic lesions, and IL-17A mRNA was also absent in peripheral blood granulocytes isolated from subjects with psoriasis (Fig.3c, d). Quantification of cytokine mRNA in skin biopsies showed significant reductions of IL-17A and IL-17F mRNA by Week 2, but only limited effects on TNF-*α* mRNA levels (Fig.2g), confirming earlier findings 4,14. Consistent with the known effects of IL-17 on epidermal chemokine production 12, blockade with secukinumab rapidly reduced the mRNA expression of keratinocyte-derived neutrophil chemoattractants such as GRO-*α* (*CXCL1*) and IL-8 (*CXCL8*) by Week 2 (Fig.2h). No consistent effects on histological or molecular parameters were observed in the placebo group.

In light of the rapid clearance of neutrophils after infusion of secukinumab, which occurred in parallel to the improvement of epidermal changes and clinical signs of psoriasis, it was speculated that reoccurrence of these cells may contribute to early clinical relapse. The finding that epidermal and dermal neutrophils, as well as neutrophil-attracting chemokines, became detectable in the low-dose cohort in about a third of the Week-12 biopsies in association with an increase in clinical disease activity between Weeks 10 and 12 supported this hypothesis (Figs1 and 2c, d, h). Indeed, subjects in the low-dose group with detectable epidermal microabscesses at Week 12 had a shorter time to relapse than those without epidermal neutrophils (14.0 vs 28.0 weeks; *P *=* *0.04; Table1). Thus, the early clinical response to secukinumab was linked to the disappearance of cutaneous neutrophils, while their reoccurrence preceded the clinical relapse observed when the effect of a single induction dose of the drug decreased.

**Table 1:** Time-to-relapse analysis in secukinumab 1 × 3 mg/kg cohort based on presence or absence of Munro’s microabscesses (MM) on histological analysis of biopsies of psoriatic lesions at Week 12

	Patients, *n* (% of total)	Lost to follow-up, *n* (% of total)	No relapse^1^ reported through Week 56, *n* (% of total)	Median time to PASI relapse, week^2^
MM at Week 12	10 (34.5)	4 (13.8)	1 (3.4)	14
No MM at Week 12	19 (65.5)	3 (10.3)	1 (3.4)	28

1 Relapse criteria were met when subject experienced loss of ≥50% of maximum Psoriasis Area and Severity Index (PASI) response or was lost to follow-up; subjects lacking a Week-12 biopsy were excluded.

2 Nonparametric Wilcoxon rank-sum test (2-sided) on time-to-relapse data yielded a *P*-value of 0.04 (corrected for ties).

## Discussion

In this study, the intravenous treatment of subjects with moderate-to-severe plaque psoriasis with the anti-IL-17A antibody secukinumab was used as a model to better understand disease mechanisms and to further dissect the response to IL-17A-targeting therapies. Treatment with secukinumab led to dose-dependent improvements of psoriasis during a 12-week period, with PASI75 (PASI90) response rates of 40% (10%), 75% (54.2%) and 82.6% (75.9%) achieved in the low-, mid- and high-dose cohorts, respectively. Particularly in the high-dose cohort, this effect was long-lasting (no relapse in half of the patients through Week 40 with the last dose received at Week 4). The clinical efficacy and safety profile observed was consistent with the findings of two phase 3 trials [FIXTURE (Full year Investigative eXamination of secukinumab vs eTanercept Using two dosing Regimens to determine Efficacy in psoriasis) and ERASURE (Efficacy of Response And Safety of two fixed secUkinumab REgimens in psoriasis)] using subcutaneous doses of secukinumab 15. In combination with the results of other trials exploring antibodies directed against IL-17A 5 or the IL-17 receptor A chain 7, these studies document the potential of IL-17 blockade as a new therapeutic approach in moderate-to-severe plaque psoriasis.

Current concepts used to explain the efficacy of anti-IL-17 therapies favour a T-lymphocytic immune response and the crosstalk between dendritic cells and Th17 cells as the driving elements in psoriasis 2. Th17 cells produce, among other cytokines, different members of the IL-17 family, such as IL-17A and IL-17F, which are overexpressed in active plaques 16, and their differentiation and maturation is thought to critically depend on IL-23 17. Within this paradigm, the principal interpretation of the high levels of clinical improvement of psoriasis in response to treatment with ustekinumab (which targets the p40 molecule shared by IL-12 and IL-23), as well as of the emerging data indicating even higher response rates for antibodies against IL-17A (blocking IL-17A homodimers and IL-17A/F heterodimers) and IL-17 receptor A (potentially blocking IL-17A, IL-17AF, IL-17F, IL-17C and IL-17E), has been that these therapies antagonize major effector cytokines related to the IL-23/Th17 axis 18. Until now, this view has remained largely unchallenged despite the identification of other cellular sources of IL-17, in particular mast cells and neutrophils 10,11,19. Neutrophils have not been a primary focus of recent models of psoriasis pathophysiology, although they represent a numerically dominant and characteristic component of the inflammatory infiltrate in this disease. In fact, the epidermal accumulation of neutrophils and formation of Munro’s microabscesses are histological hallmarks of psoriasis and likely reflect the presence of innate immune mechanisms within the psoriasis inflammatory cascade.

Based on the investigation of large numbers of biopsies, the present study confirms earlier findings of smaller studies 10,11,19 that the number of IL-17-containing T cells in active psoriasis is small (<10% of CD3-positive cells) and that neutrophils, especially when Munro’s microabscesses are present, are the numerically dominant cell type with IL-17 detectable by immunohistochemistry.

There has been some discussion of whether neutrophils only release preformed IL-17 or actually synthesize IL-17 mRNA and protein in the skin. In the present study, IL-17A mRNA was detected in T cells, but not in neutrophils isolated from psoriatic plaques (Fig.3d); thus, these findings would favour an IL-17 protein storage model. Expression of IL-17A mRNA in conjunction with ROR*γ*t co-expression and release of IL-17 by extracellular trap formation was, however, observed in epidermal neutrophils in a recent investigation of 2 models of human skin inflammation 20. The ability of neutrophils to produce IL-17A mRNA and protein under control of ROR*γ*t in response to IL-6 and IL-23, both of which are overexpressed in psoriatic plaques 21, was also demonstrated in another study investigating human and mouse neutrophils 22. Based on these different findings, it is not fully clear at present whether IL-17 found in cutaneous neutrophils in active psoriasis is newly synthesized *in loco* or is contained preformed in neutrophils entering the skin, similar to what has been observed for other neutrophil-derived mediators such as TNF-*α* 23,24. While available data from other studies and the data presented here suggest that neutrophils are a relevant source of IL-17 in psoriasis, further work is required to clarify whether, and at which stage of their development, or under which conditions, the synthesis of IL-17 in neutrophils actually occurs.

Based on our findings on the kinetics of changes in T cells, CD11c-positive dendritic cells, neutrophils and keratinocytes, as well as the expression of key cytokines and chemoattractive mediators following treatment with secukinumab, some relevant conclusions can be drawn: The most prominent observation 2 weeks after a single infusion of the anti-IL-17A antibody secukinumab was a significant reduction in psoriatic epidermal abnormalities (hyperparakeratosis, acanthosis and hyperproliferation), together with strongly decreased mRNA expression levels of keratinocyte-derived chemokines GRO-*α* (*CXCL1*) and IL-8 (*CXCL8*) and an almost complete clearance of epidermal IL-17-positive neutrophils. Secukinumab may therefore interfere with the influx of neutrophils into psoriatic lesions indirectly by abrogating the effect of IL-17A on keratinocytes and other cells (e.g. endothelial cells) involved in neutrophil recruitment 25. In addition, secukinumab may target direct effects of IL-17A on neutrophil survival and activation, as previously described 22,26. Early normalization of the epidermal microarchitecture could help explain the magnitude of the clinical improvement observed at Week 2, with ∼60% of subjects treated with the highest dose of secukinumab achieving a PASI75 response at that time point; a similar response pattern was observed for the anti-IL-17A antibody ixekizumab 5. It should also be noted that there was a substantial early reduction of mRNA expression levels of IFN*γ*, and especially IL-17A and F, but not of TNF-*α* mRNA (Fig.2g). As we could detect IL-17A mRNA only in T cells isolated from psoriatic lesions, which are also regarded as the main source of IFN*γ*, a possible explanation is an early inhibition of T-cell cytokine production by anti-IL-17A treatment.

At Week 12, secukinumab had also reduced the number of lesional CD11c-positive dendritic cells and T cells similar to what has been proposed to coincide with more final disease resolution during treatment with other targeted therapies for psoriasis 3. Following the early effects on epidermal changes, neutrophil influx and cytokine synthesis, this normalizing effect of secukinumab on relevant cells and mediators of adaptive skin immunity likely contributes to the observed sustainability of the clinical response, with disease control maintained in many subjects for >30 weeks after the last infusion of the drug.

Taken together, the results of the present study strengthen the view that neutrophils are a potential source of IL-17 in psoriasis and newly identify these cells as an early cellular target of the novel class of IL-17-directed therapies. Although blockade of IL-8 and neutrophil apheresis have shown some effects in pustular variants of psoriasis 27,28, it is unlikely that targeting of neutrophils or single neutrophil-chemotactic factors alone will be a successful therapeutic approach in plaque-type psoriasis. We propose a model (Fig. S7) in which the early response to anti-IL-17A antibodies such as secukinumab involves the interruption of a neutrophil–keratinocyte crosstalk in which IL-17 derived from T cells, and potentially neutrophils, stimulates the epidermal production of chemokines that, in turn, orchestrate the further influx of neutrophils into psoriatic lesions, while the full and long-term clinical response is associated with the reduction of lesional dendritic cells and T cells. The observed rapid epidermal clearance of neutrophils is considered to be an important element of IL-17A inhibition in psoriasis as these cells – through the release of mediators such as TNF-*α*, LL-37 and IL-17A – perpetuate and enhance the abnormal defense programme characteristic of the disease 29–31.

## Abbreviations

AE: adverse event
IL: interleukin
PASI: Psoriasis Area and Severity Index
Th17: T-helper type-17 cell subset
Tregs: regulatory T cells

## Supporting Information

**Figure S1.** Subject disposition. **Figure S2.** Images depicting semi-quantitative assessment categories. **Figure S3**. Study design. **Figure S4.** Assessment of peripheral blood T-lymphocyte subsets. **Figure S5.** Examples of control stainings in immunohistochemistry and immunofluorescence. **Figure S6.** Assessment of additional epidermal and cellular markers over time. **Figure S7.** Proposed simplified model of psoriasis pathogenesis and early effects of anti-IL-17 therapy. **Table S1.** NanoString nCounter Gene Expression Maestro CodeSet probe sequences for genes reported in this study. **Table S2.** Demographic and Baseline clinical characteristics of study subjects.
