# Supplementary material for: Evidence that a neutrophil–keratinocyte crosstalk is an early target of IL-17A inhibition in psoriasis
Source: Exp Dermatol. 2015 May 8;24(7):529–35. doi: 10.1111/exd.12710 (PMC4676308; doi:10.1111/exd.12710)
Supplement: Supplementary file 1 [file exd0024-0529-sd1.doc]

**Evidence That a Neutrophil-Keratinocyte Crosstalk is an Early Target of IL-17A Inhibition in Psoriasis**

Kristian Reich^1^, Kim A. Papp^2^, Robert T. Matheson^3^, John H. Tu^4^, Robert Bissonnette^5^, Marc Bourcier^6^, David Gratton^7^, Rodion A. Kunynetz^8^, Yves Poulin^9^, Les A. Rosoph^10^, Georg Stingl^11^, Wolfgang M. Bauer^11^, Janeen M. Salter^12^, Thomas M. Falk^1^, Norbert A. Blödorn-Schlicht^1^, Wolfgang Hueber^12^, Ulrike Sommer^12^, Martin M. Schumacher^12^*, Thomas Peters^12^, Ernst Kriehuber^12^, David M. Lee^12^, Grazyna A. Wieczorek^12^, Frank Kolbinger^12^ and Conrad C. Bleul^12^

^1^Dermatologikum Hamburg and SCIderm Research Institute, Hamburg, Germany; ^2^Probity Medical Research Inc, Waterloo, Ontario, Canada; ^3^Oregon Medical Research Center PC, Portland, OR, USA; ^4^Skin Search of Rochester, NY, USA; ^5^Innovaderm Research Inc, Montreal, Quebec, Canada; ^6^Dermatology Clinic, Moncton, New Brunswick, Canada; ^7^International Dermatology Research, Montreal, Quebec, Canada; ^8^Ultranova Skincare, Barrie, Ontario, Canada; ^9^Centre de Recherche Dermatologique du Québec Métropolitain, Quebec City, Quebec, Canada; ^10^North Bay Dermatology Centre, North Bay, Ontario, Canada; ^11^Department of Dermatology, Division of Immunology, Allergy and Infectious Diseases, Medical University of Vienna, Austria; ^12^Novartis Institutes for BioMedical Research, Basel, Switzerland.

*Currently employed by Firalis SAS, Huningue, France.

*Correspondence*: Kristian Reich, Dermatologikum Hamburg, Stephansplatz 5, 20354 Hamburg, Germany, Tel.: +49 40 351075-79, Fax: +49 40 351075-40, e-mail: kreich@dermatologikum.de

**Materials and methods**

**Sample size calculation for the phase 2 trial**

The sample size was based on the co-primary analysis of the proportion of subjects who had not relapsed at any time up to and including Week 56. With complete data from 30 subjects in each active treatment group, this study would have approximately 79% power to show a statistically significant difference between a pair of active treatment groups. This calculation was based on the assumption that the true proportions of subjects who had not relapsed in the 2 treatment groups being compared were 30% and 65%, respectively. Model predictions from a prior pharmacokinetics/pharmacodynamics study suggested that 30% is the expected proportion of subjects who would not have relapsed in the 1 x 3 mg/kg group. A 1-sided 5% alpha level was assumed in the calculation. The total sample size did not include any additional subjects to allow for drop-outs and/or incomplete data. All subjects who dropped out were considered to have relapsed.

**Randomization and blinding for the phase 2 trial**

The randomization scheme was generated by Novartis Drug Supply Management using a validated system. The randomization scheme was reviewed and approved by the Biostatistics Quality Assurance group of Novartis and was locked after approval. Subjects were assigned randomization numbers, according to the randomization schedule. Each site, upon evaluation of a qualified subject for the trial, faxed the enrollment sheet to the clinical trial leader (CTL) at the fax number provided. The CTL then assigned the randomization number in a sequential manner and faxed it back to the unblinded pharmacist or qualified site personnel at the site, who then prepared and provided the study medication for the clinic in a blinded fashion. This enabled a single randomization code to be implemented across multiple sites. One center was prematurely terminated and data from this site could not be used for analysis. Therefore, additional subjects were recruited to replace the 30 subjects randomized at this center. These replacement subjects were assigned randomization numbers according to the replacement randomization list. From this replacement list, only randomization numbers with the same last 3 digits as the subjects from this center were used. Within this subset of replacement randomization numbers, the numbers were assigned sequentially (e.g. 5101was replaced by 6101, 5102 with 6102 and so on).

Treatment allocation and clinical assessment of the subjects were blinded. For preparation of the study medication from bulk supplies, treatment allocation cards were sent to the pharmacist or qualified site personnel at the investigator’s site. To maintain the blind of the study, the appearance of placebo infusion bags, ready to administer to the subject, was identical to that of active drug infusion bags. Placebo and active medication were prepared by an unblinded pharmacist or qualified site personnel assigned at each site. Randomization data were kept strictly confidential and were accessible only to authorized personnel until unblinding of the trial after database lock. At the conclusion of the study, the occurrence of any emergency code breaks was determined after return of all code break reports to Novartis. Only when the study was completed and the data file verified were the drug codes to be broken and made available for data analysis. Since this was an exploratory study, an Exploratory Analysis Team was assigned. This team had access to the randomization codes at the time of the interim analyses for the purpose of (i) conducting the interim analysis and (ii) planning future trials or amendments to the design of the current trial. The Exploratory Analysis Team included the trial statistician, trial programmer, modeling and simulation (M&S) modeler, and M&S programmer. The M&S modeler and programmer used the data as part of an integrated model of all the phase 2 data to inform the dose and regimen for the phase 3 program. Unblinding was only permitted in the case of subject emergencies and at the conclusion of the study. To maintain data integrity, no subject-level data were circulated; therefore, blinding was maintained at the individual subject level. In addition, the bioanalysts involved in the measurement of the mRNA and flow cytometry samples received a copy of the randomization schedule to facilitate sample analysis. The bioanalysts provided data to the team under blinded conditions. The pharmacist or qualified site personnel and bioanalyst kept this information confidential until clinical database lock.

**Limitations of the clinical portion of the phase 2 trial**

Limitations of this exploratory study included the small sample size, which did not permit definitive conclusions regarding the clinical efficacy or safety of secukinumab in treating psoriasis. In addition, the imbalance in subject numbers and time of follow-up between the secukinumab and placebo groups is a potential confounding factor in the interpretation of adverse event rates.

**RNA extraction, NanoString nCounter® and quantitative reverse-transcription polymerase chain reaction gene-expression analyses of skin biopsies**

Approximately 750 µm of cryosection of each optimal cutting temperature‒embedded psoriasis skin biopsy were used for RNA isolation, each disrupted (TissueRuptor, Qiagen NV, Venlo, the Netherlands) and homogenized in 400 µL buffer RLT (Qiagen). Subsequent RNA isolation steps were performed according to the manufacturer’s instructions. Dithiothreitol (SERVA Electrophoresis, Heidelberg, Germany) was used instead of β-mercaptoethanol, and a proteinase-K digestion of the lysate was included, as well as an on-column DNase digestion. Finally, RNA was eluted in 25 µL of RNase-free water. Elution of RNA was repeated using the first eluate. RNA samples were stored at –80°C.

For the NanoString nCounter gene-expression analysis (NanoString Technologies, Seattle, WA, USA), 50 ng of RNA was pre-amplified using the SensationPlus^TM^ FFPE Amplification Kit (Affymetrix, Santa Clara, CA, USA), and 250 ng of the pre-amplified sense RNA was then hybridized with the nCounter Gene Expression CodeSet Maestro (NanoString Technologies) at 65°C for 16 h. In addition, 1 µL of unlabeled standard desalted oligonucleotide for *B2M (β2-microglobulin)*, *KRT10* (*keratin-10*), *S100A7*, *S100A8* and *S100A9* (each at 14.29 nM; sequences shown in Table S1) was spiked into the hybridization master mix to compete with respective labeled reporter probes and prevent saturation of the flow-cell imaging surface by these highly expressed genes. Post-hybridization processing procedures were performed as recommended by NanoString Technologies. Cartridges were scanned at a resolution of 600 fields of view. Gene-expression barcode counts were analyzed using the nSolver™ Analysis Software v1.0 (NanoString Technologies). Raw NanoString barcode counts for each gene underwent 2 subsequent normalization steps. First, a technical lane-to-lane normalization was performed using the geometric mean of counts obtained for 6 spike-in alien positive control probe sets enclosed in the CodeSet. Second, a biological messenger RNA content–related normalization (overriding the preceding technical normalization) was performed using the geometric mean of the technically normalized counts of the 3 reference genes [*RPL13A*, *RPL19* and *ubiquitin C* (*UBC*)]. The two gender-specific transcripts, RPS4Y1 (Y-chromosome) and XIST (X-chromosome), were used to control for the correct gender assignment at the molecular level for the subjects enrolled in this study. The accuracy of the assignment of the gender of a sample’s donor is a good surrogate to assess the overall accuracy of the process (2).

To provide controls for the data obtained by the NanoString nCounter gene-expression analysis, cutaneous expression levels of *IL17A (interleukin-17A)* and *IFNG* (*interferon-γ*) were also determined by quantitative reverse-transcription polymerase chain reaction (PCR). To this end, complementary DNA was synthesized using the Applied Biosystems® High Capacity cDNA Reverse Transcription Kit (Life Technologies, Grand Island, NY, USA). Samples were processed with a cDNA (total RNA equivalent) input of 12.5 ng/reaction. All samples were measured in duplicate using a Bio-Rad iCycler Thermal Cycler with iQ5 Multicolor Real-Time PCR Detection System (Bio-Rad Laboratories, Hercules, CA, USA). The Real-Time program was 95°C for 10 min (1 cycle), 95°C for 15 s and 60°C for 60 s (50 cycles); the threshold was 0.13. The following Applied Biosystems TaqMan® Assays (Life Technologies) were used: *UBC* (*ubiquitin C*) – Hs00824723_m1; *B2M* – Hs99999907_m1; *IFNG* – HS00174143_m1; and *IL17A* – HS00936345_m1. The quantitative reverse-transcription PCR results were evaluated using the iQ5 Optical System Software, Version 2.0 (Bio-Rad Laboratories). Quantification was based on ΔΔCT calculations. Samples were normalized to the geometric mean of the relative quantities of the 2 reference genes, *B2M* and *UBC.* Comparison of the expression data for *IL17A* and *IFNG* by NanoString nCounter gene-expression and quantitative reverse-transcription PCR analyses revealed an excellent correlation between the 2 datasets, with Spearman correlation coefficients of 0.87 for *IL17A* and 0.69 for *IFNG* (*P* < 0.000001 for both).

**Immunohistochemistry and immunofluorescence**

Primary reagents for immunohistochemical detection were anti-human CD3 antibody (monoclonal mouse, clone F7.2.38, M7254; Dako, Glostrup, Denmark), anti-CD11c antibody (rabbit monoclonal, clone EP1347Y, ab52632; Abcam®, Cambridge, United Kingdom), anti–IL-17 antibody (polyclonal goat, reacting mainly with IL-17A and minimally with IL-17F; AF-317-NA; R&D Systems, Minneapolis, MN, USA), anti-human myeloperoxidase (polyclonal rabbit, A0398; Dako), anti-human mast cell tryptase (monoclonal mouse, clone AA1, M7052; Dako) and anti-human Ki67 antigen (monoclonal mouse, clone MIB-1, M7240; Dako). For detection of IL-17A, the REAL^TM^ biotinylated secondary antibody (AB2; see Materials and methods section in main article) was replaced by biotinylated Polyclonal Anti-Swine, Goat, Mouse, Rabbit Immunoglobulins, Multi-Link (Dako). Representative examples of the prospectively defined semi-quantitative scoring system used to evaluate Ki67, CD11c, CD3, dermal IL-17A, myeloperoxidase and mast cell tryptase are shown in Fig. S2. Primary antibodies for immunofluorescence labeling were anti-CD3 (monoclonal rabbit, clone SP7, RM-9107-S; NeoMarkers, Lab Vision Corporation, Fremont, CA), anti-myeloperoxidase (rabbit polyclonal, ab45977; Abcam), anti-human MCT (Dako) and anti–IL-17 antibody (AF-317-NA; R&D Systems). Secondary reagents used to visualize the reaction were donkey anti-goat Alexa Fluor® 488 (A-11055; Molecular Probes®, Life Technologies) and chicken anti-mouse or anti-rabbit Alexa Fluor® 594 (A-21201/A-21442, Molecular Probes). For control purposes, specific primary reagents were replaced by isotype-matched polyclonal or monoclonal immunoglobulins of the same species; anti–IL-17 polyclonal antibody was also pre-absorbed with recombinant human (rh)IL-17A and F (Fig. S5).

**Analysis of peripheral blood T cells and isolated peripheral blood and skin leukocyte subsets**

T-lymphocyte subsets were determined after *in vitro* stimulation of peripheral blood mononuclear cells for 4 h using a leukocyte-activation cocktail containing phorbol-12-myristate-13-acetate, ionomycin and brefeldin A (Becton, Dickinson and Company, Franklin Lakes, NJ, USA). CD4 T cells were determined using anti-CD3, PerCP (Becton, Dickinson; clone SK7) and anti-CD4 PE-Cy7 (Becton, Dickinson; clone SK3) antibodies. T-helper type-17 cells were determined in the CD4-positive T-lymphocyte population by intra-cellular staining of IL-17A using anti-human IL-17A Alexa Fluor^®^ 647 (eBioscience, San Diego, CA, USA; clone eBio64DEC17). T-helper type-1 cells were determined in the CD4^+^ T-lymphocyte population by intra-cellular staining using anti-human interferon-γ Alexa Fluor^®^ 488 (Becton, Dickinson; clone B27). Regulatory T cells were assessed in unstimulated peripheral blood mononuclear cells using a combination of anti-human CD4, CD25, and CD127 monoclonal antibodies (Becton, Dickinson; clones SK3, 2A3, hIL-7R-M21) and a phycoerythrin mouse anti-human FoxP3 antibody (Becton, Dickinson; clone 259D/C7).

T lymphocytes, monocytes and granulocytes were purified from peripheral blood by anti-CD3, -CD14 or -CD15 MicroBeads (Miltenyi Biotec, Bergisch Gladbach, Germany), following the manufacturer’s recommendations. The purity of resulting cell populations was confirmed by fluorescence-activated cell-sorting analysis using antibodies against CD45, CD15, CD3 and CD14, and by quantitative PCR for respective lineage antigens. Cell suspensions from psoriatic plaques were obtained as described by Hutter et al. (1). In brief, biopsies were minced and immersed in collagenase IV (Worthington Biochemical, Lakewood, NJ), dissolved at 0.3% w/v in RPMI1640/10% FCS, and incubated at 37°C for 60 min. The digested tissue was further disaggregated using a cell dissociation sieve (Sigma-Aldrich, St. Louis, MO). Cells were pelleted and resuspended in ice-cold RPMI1640/10% FCS. The cell suspension was then sorted using flow cytometry to isolate the different leukocyte subsets as described above. Cells were identified using the following marker combinations: T lymphocytes: CD45^+^CD3^+^; mast cells: CD45^+^CD203c^+^; dendritic cells: CD45^+^HLA-DR^high^CD11c^+^; and neutrophils: CD45^+^HLA-DR^low/negative^CD11b^+^CD15^+^. The neutrophil population was further characterized and found to stain positive for surface CD66b and cytosolic myeloperoxidase; cell sorting was performed on a FACSAria™ cell sorter (Becton, Dickinson). All protocols for obtaining and studying human tissues and cells were approved by the relevant institutional review boards and adhered to the regulations of the Declaration of Helsinki.

Sorted cells were lysed in TRI Reagent® (Sigma-Aldrich®, St. Louis, MO, USA), and RNA was isolated according to the manufacturer's recommendations. Total RNA was reverse-transcribed using a Superscript® III First-Strand Synthesis System (Invitrogen™; Life Technologies), and quantitative PCR was performed under standard conditions with an Applied Biosystems 7500 Fast Real-Time PCR System (Life Technologies). Samples were run in duplicate for each probe, and quantification was based on ΔΔCT calculations. Samples were normalized to *B2M*. The following predesigned TaqMan probes from Applied Biosystems were used – *B2M*: Hs99999907; *CD3E*: Hs01062241_m1; *CD1c*: Hs00233509_m1; *PR3* (*proteinase 3*): Hs01597752_m1; and *IL17A*: Hs00174383_m1.

References

1. Hutter C, Kauer M, Simonitsch-Klupp I *et al.* Notch is active in Langerhans cell histiocytosis and confers pathognomonic features on dendritic cells. Blood 2012: **120**: 5199–5208.

2. Staedtler F, Hartmann N, Letzkus M, *et al*. Robust and tissue-independent gender-specific transcript biomarkers. Biomarkers 2013: **18**: 436-45.

**Figure S1**. Subject disposition and biopsy status. AE, adverse event; i.v., intravenous.


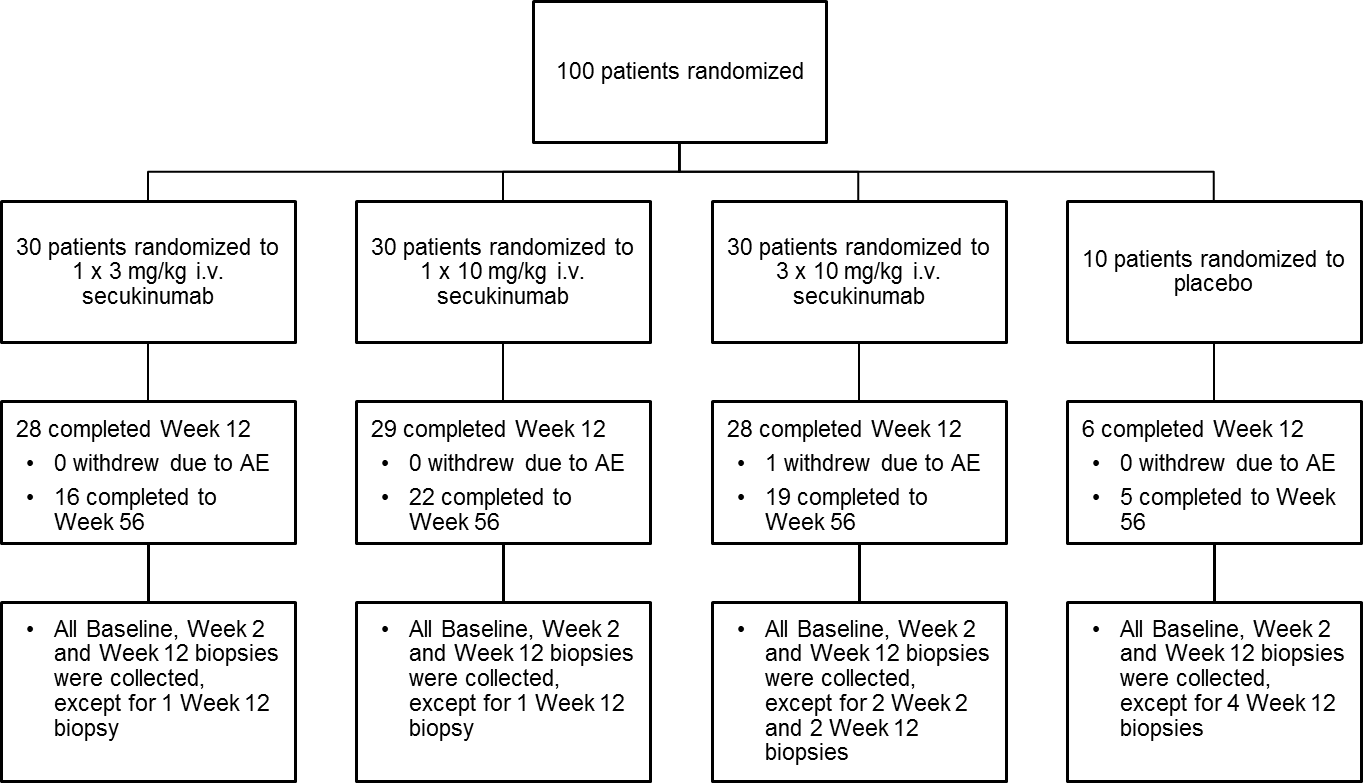


**Figure S2. Images depicting semi-quantitative assessment categories. Categories shown are CD3 total grade, CD11c total grade, IL-17A dermal grade, Ki67 epidermal grade, mast cell (MC) tryptase total grade, myeloperoxidase (MPO) dermis grade, MPO total grade, and parakeratosis (PKT). Note: Grading of interleukin (IL)-17A staining is for dermal cells only. Epidermal neutrophils always stain positive, and sometimes there is no quantitative correlation with the dermal infiltrate.**

| 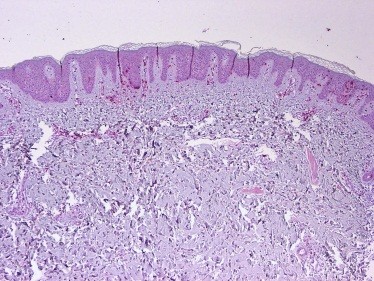  CD3 total grade 1 | 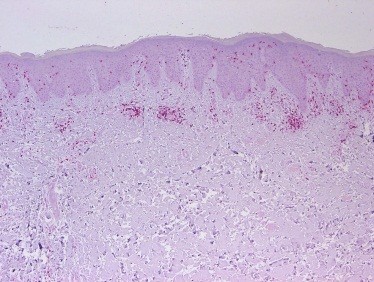  CD3 total grade 2 | 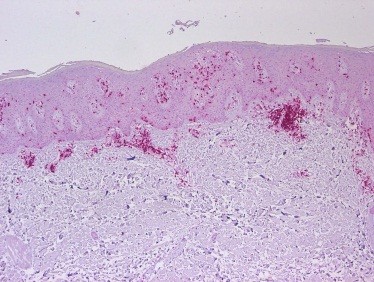  CD3 total grade 3 | 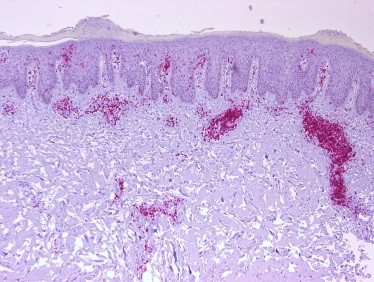  CD3 total grade 4 |  |
| --- | --- | --- | --- | --- |
| 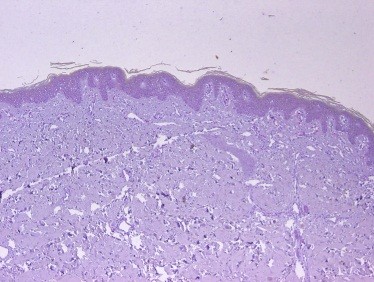  CD11c total grade 1 | 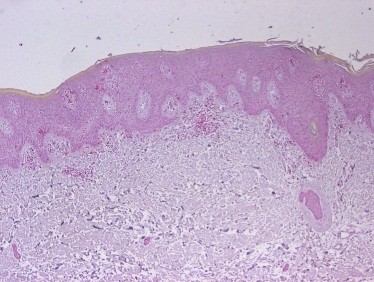  CD11c total grade 2 | 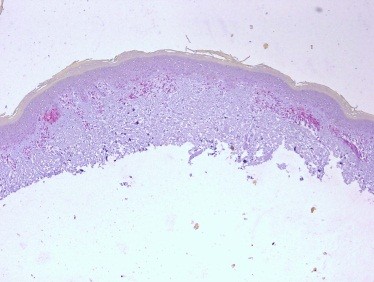  CD11c total grade 3 | 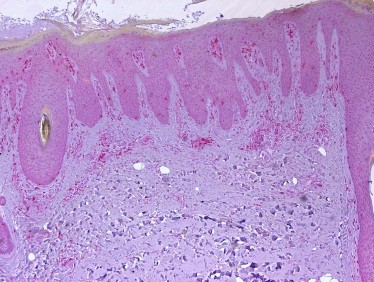  CD11c total grade 4 |  |
| 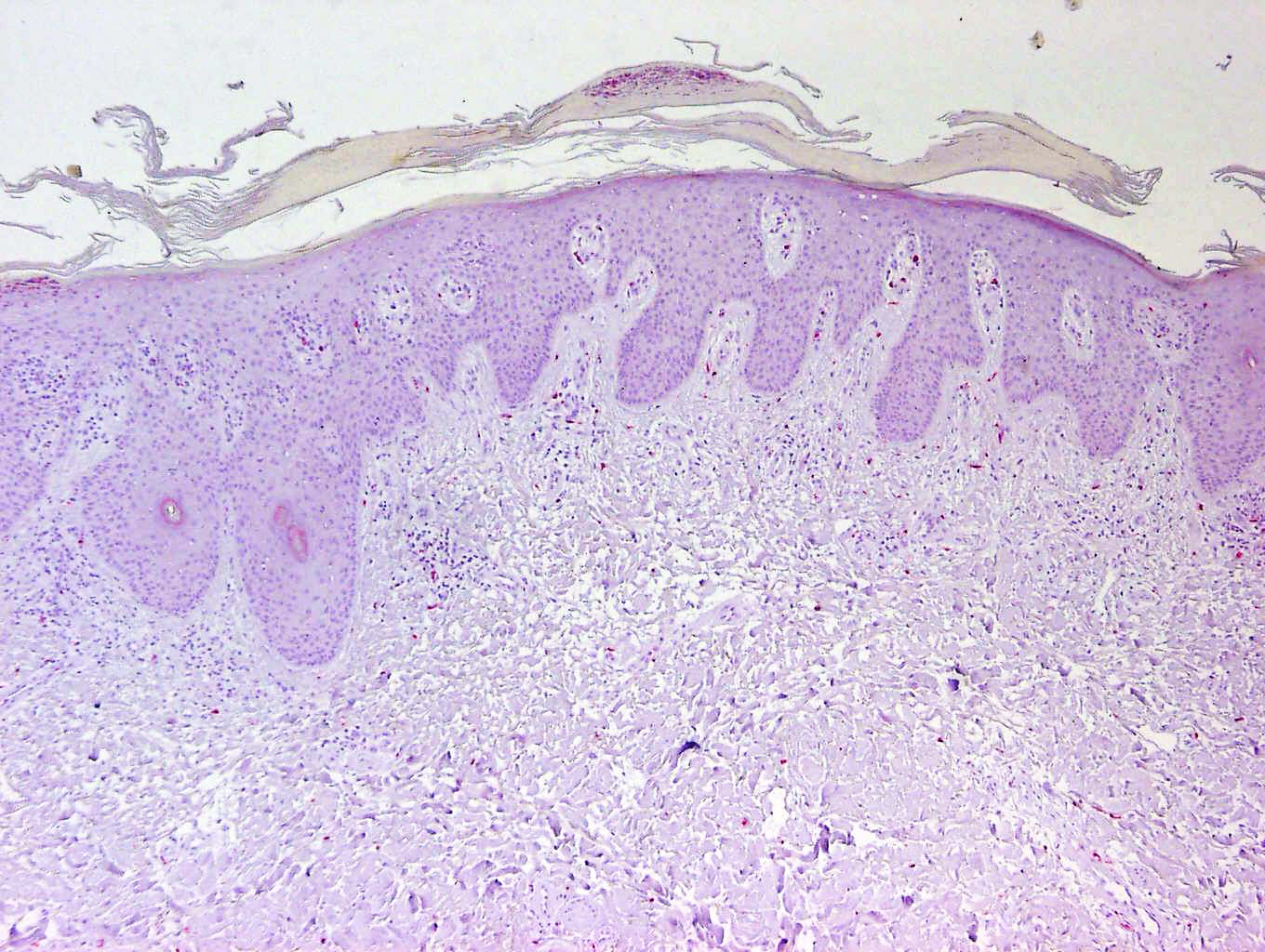  IL-17A dermal grade 1 | 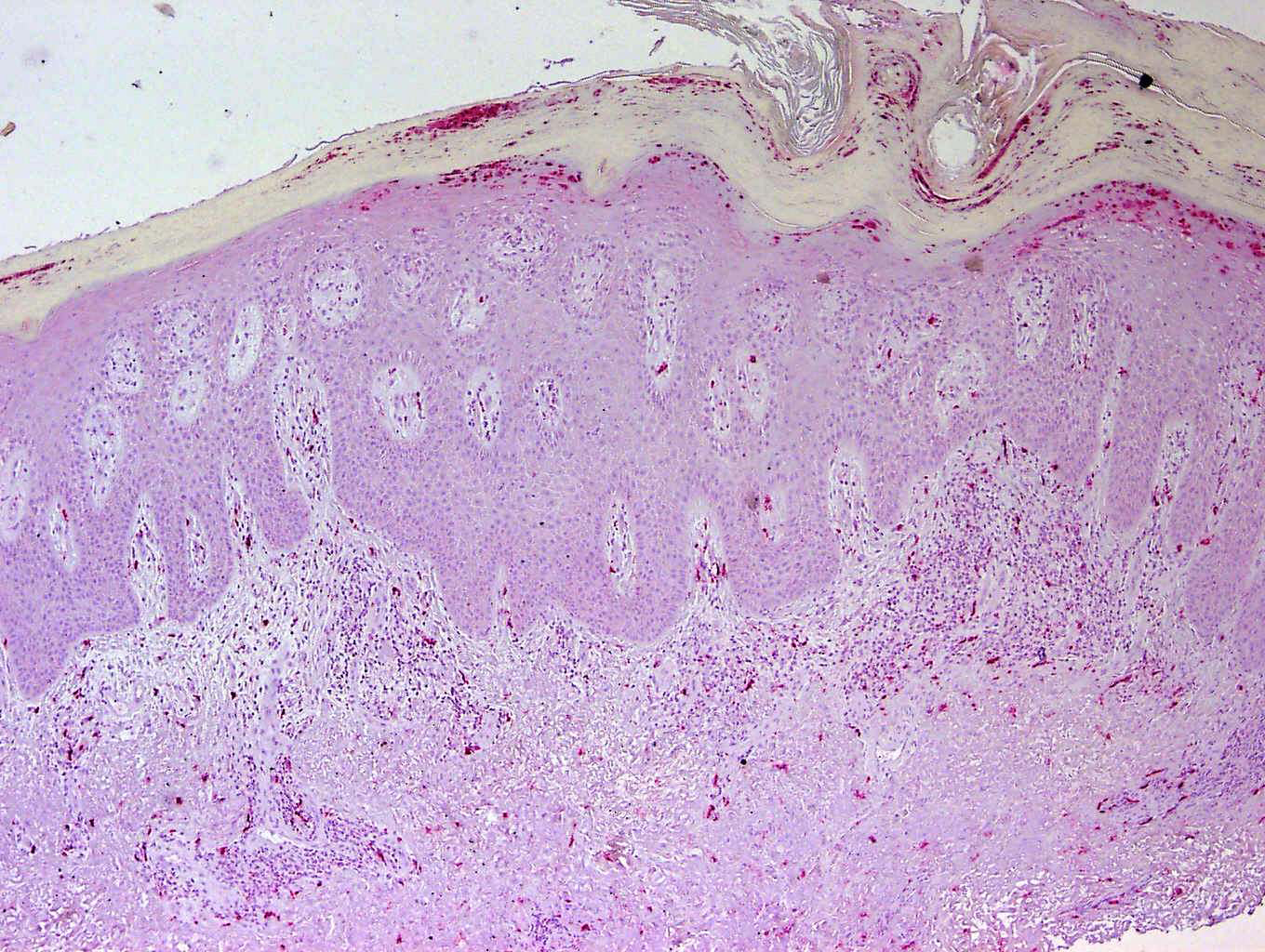  IL-17A dermal grade 2 | 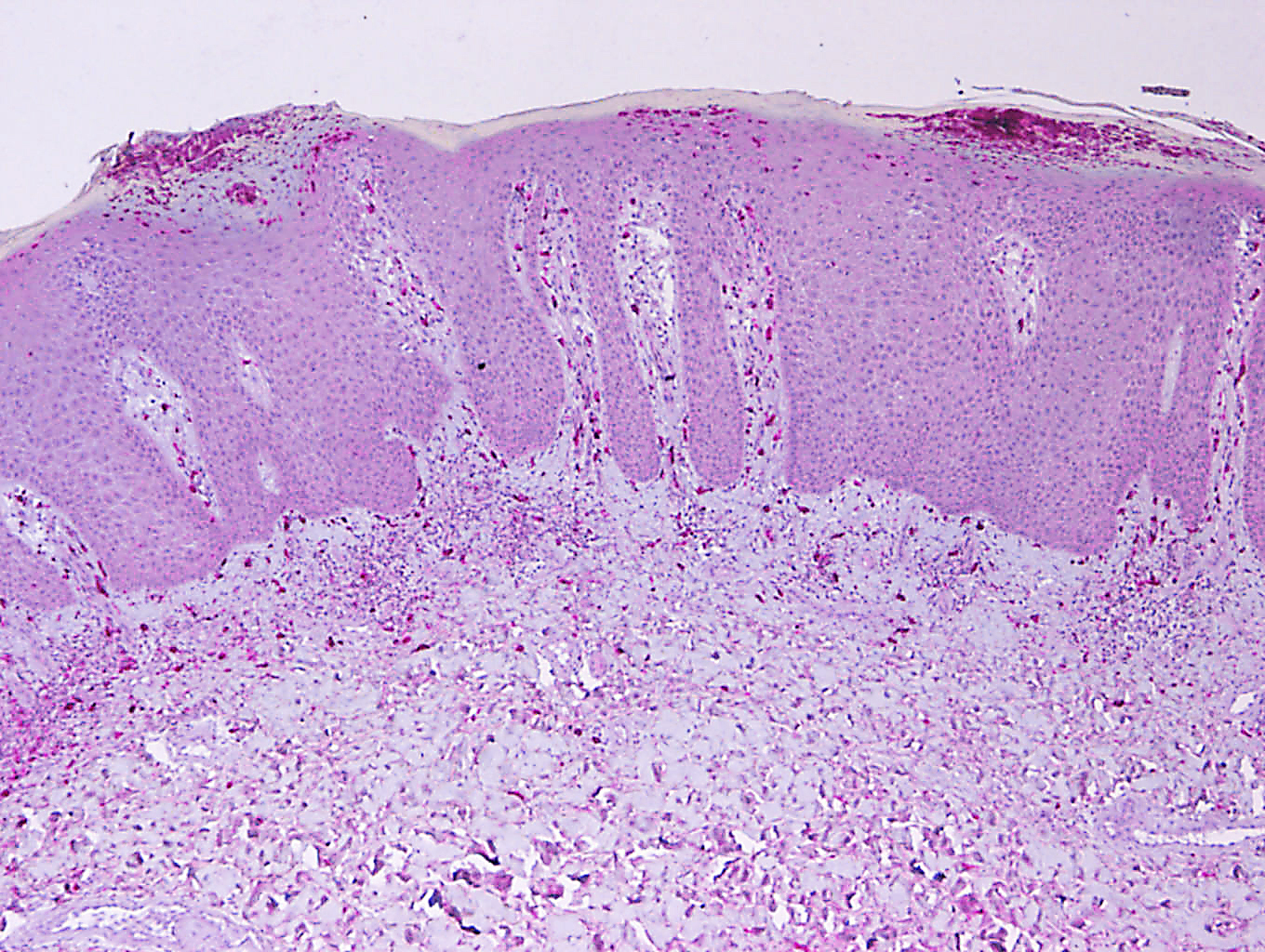  IL-17A dermal grade 3 | 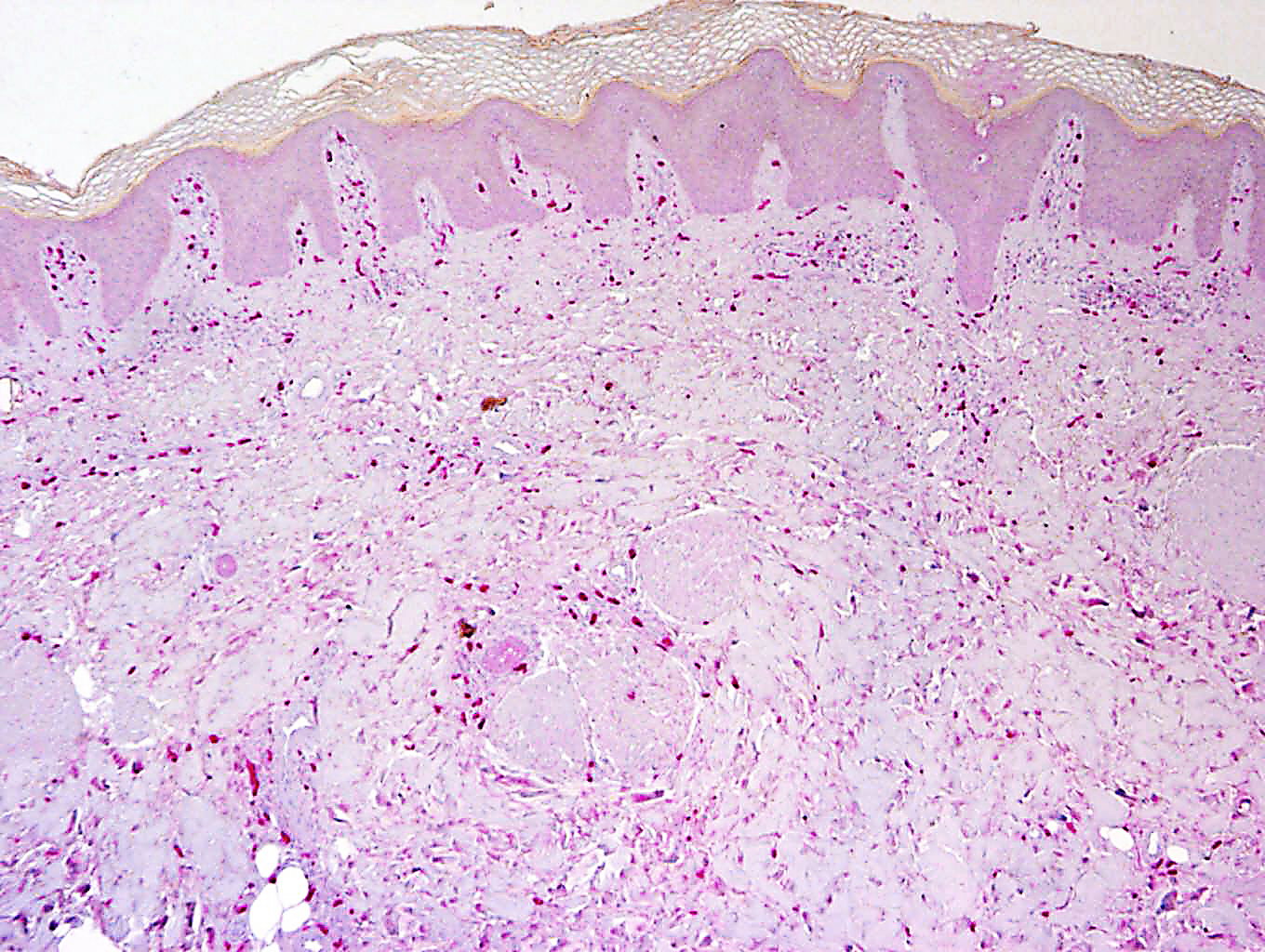  IL-17A dermal grade 4 |  |
|  | | | |  |
| 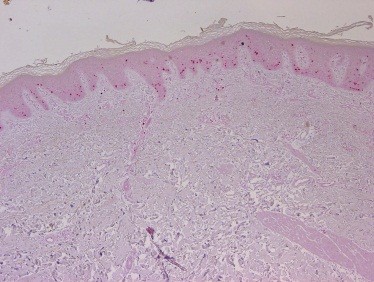  Ki67 epidermal grade 1 | 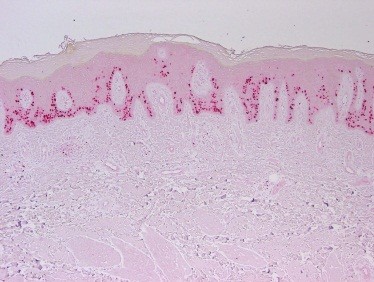  Ki67 epidermal grade 2 | 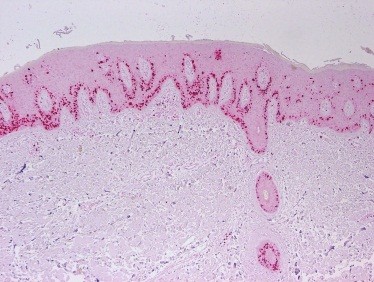  Ki67 epidermal grade 3 | 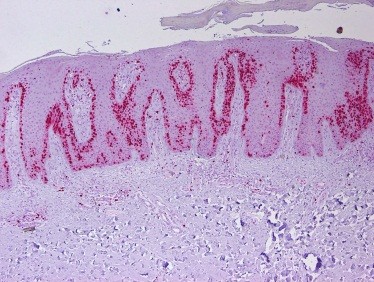  Ki67 epidermal grade 4 |  |
| 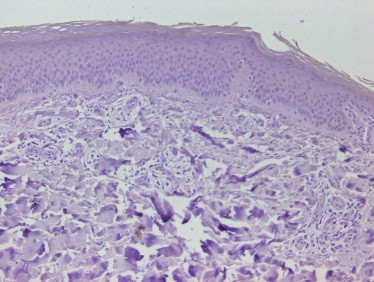  MC tryptase total grade 0 | 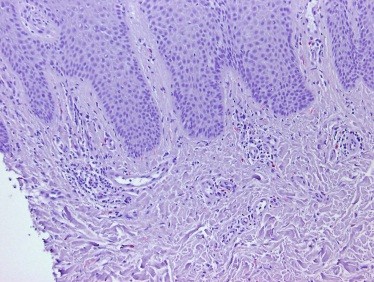  MC tryptase total grade 1 | 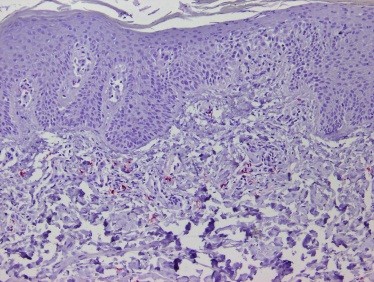  MC tryptase total grade 2 | 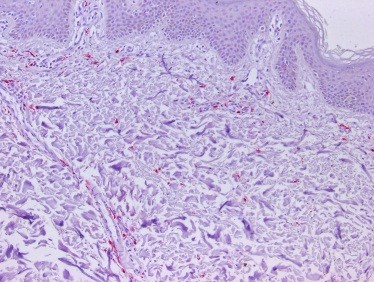  MC tryptase total grade 3 | 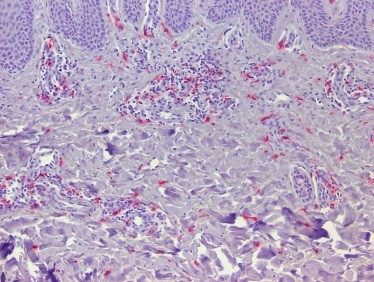  MC tryptase total grade 4 |
| 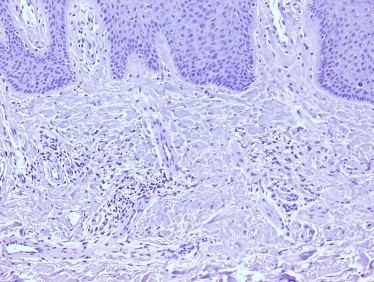  MPO dermis grade 0 | 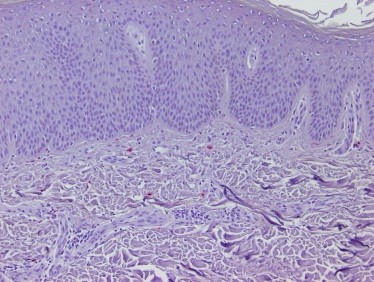  MPO dermis grade 1 | 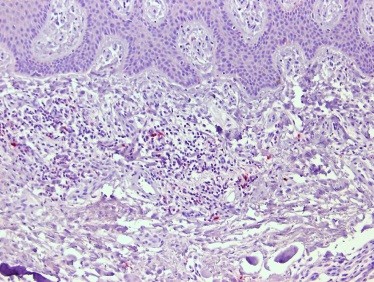  MPO dermis grade 2 | 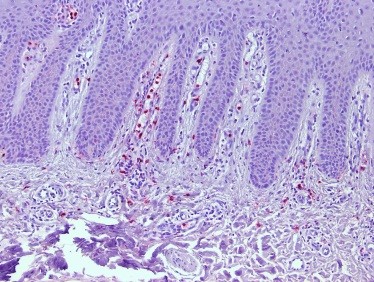  MPO dermis grade 3 | 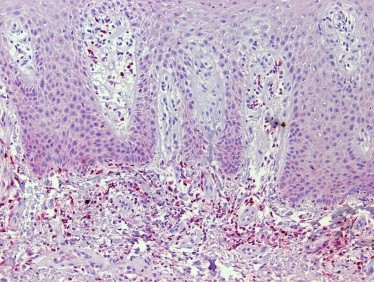  MPO dermis grade 4 |
| 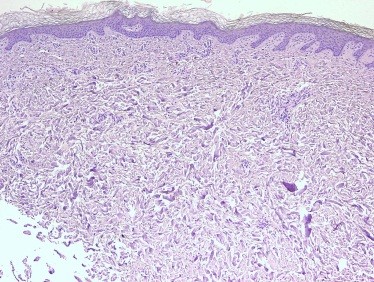  MPO total grade 0 | 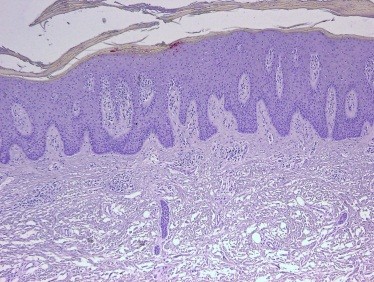  MPO total grade 1 | 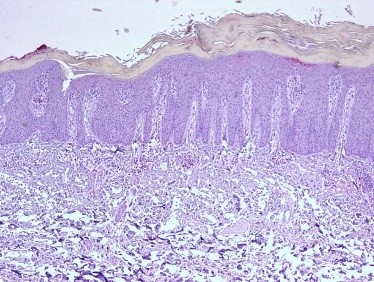  MPO total grade 2 | 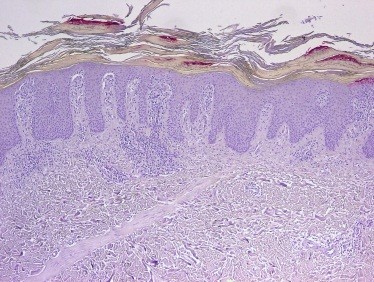  MPO total grade 3 | 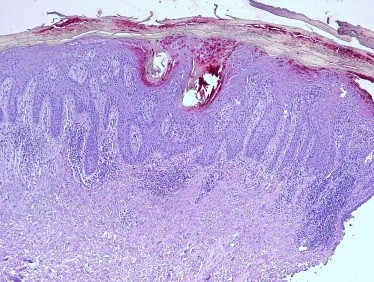  MPO total grade 4 |
| 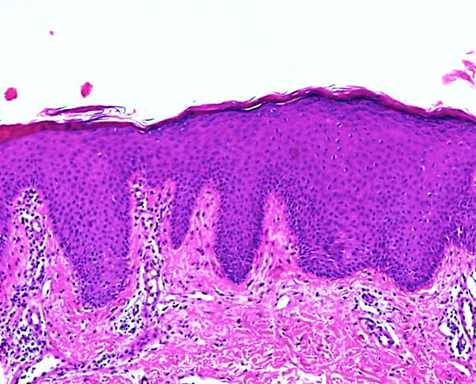  PKT grade 1 | 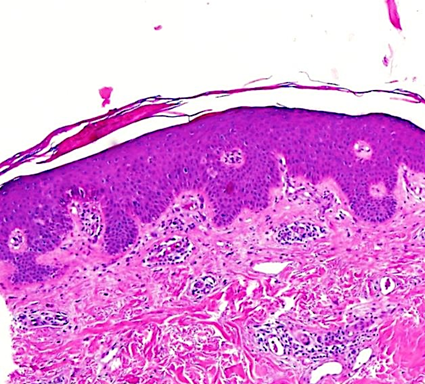  PKT grade 2 | 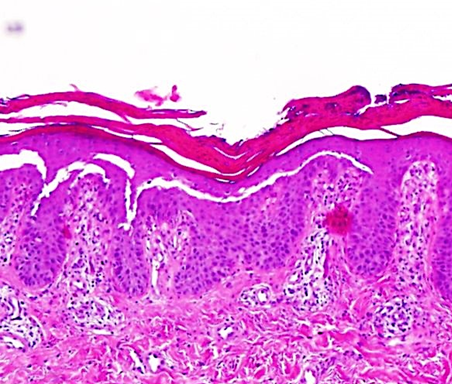  PKT grade 3 |  | |

**Figure S3**. Study design. i.v., intravenously; R, randomization.


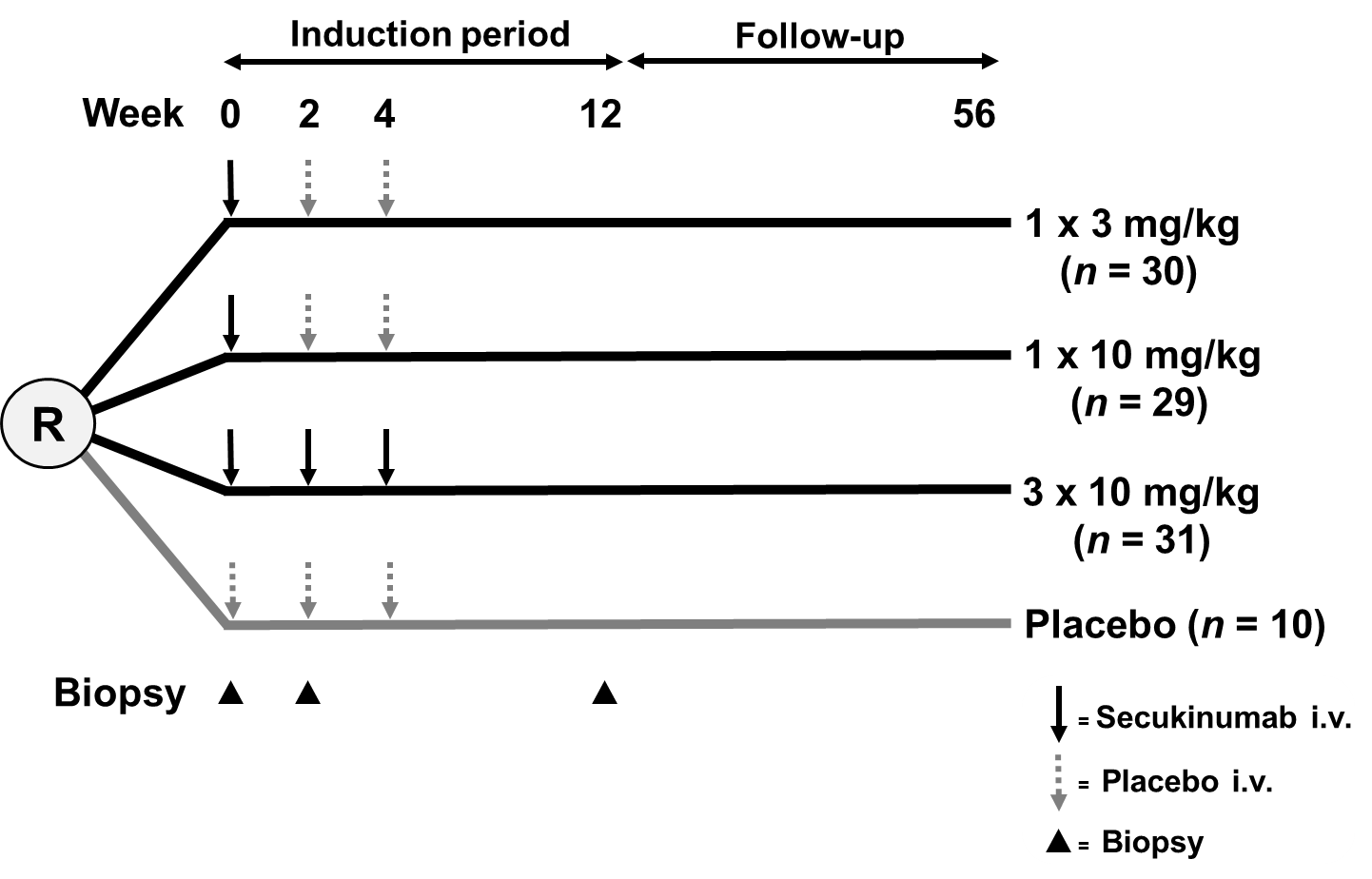


**Figure S4.** Assessment of peripheral blood T-lymphocyte subsets**.** These subsets were assessed in subjects allocated to the different treatment groups over the course of the study. Blood samples were collected at screening, on Day 1, at Weeks 2, 4, 12 and 28, and at end of study (EOS). Percentages for subsets were determined by flow cytometry in *in vitro* stimulated peripheral blood mononuclear cell samples for (a) CD4 T lymphocytes, (b) T-helper (Th) 1 and (c) Th17 subsets and (d) in unstimulated peripheral blood mononuclear cell samples for regulatory T cells (Tregs). IFN, interferon, IL, interleukin.

**
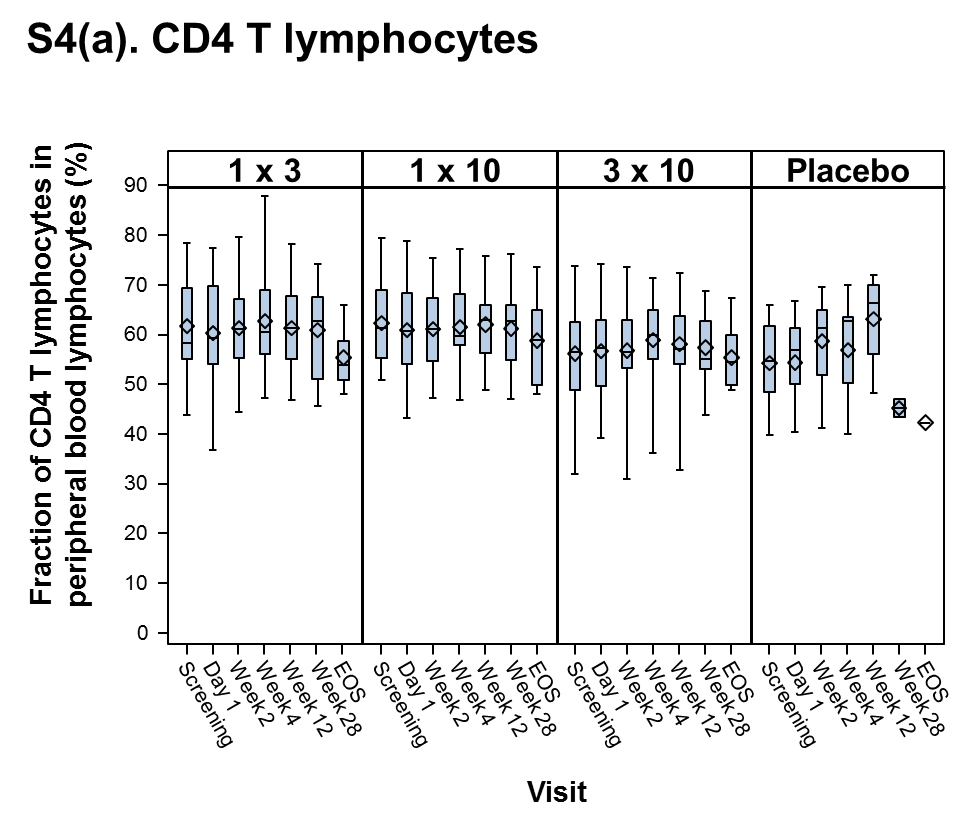
**

**
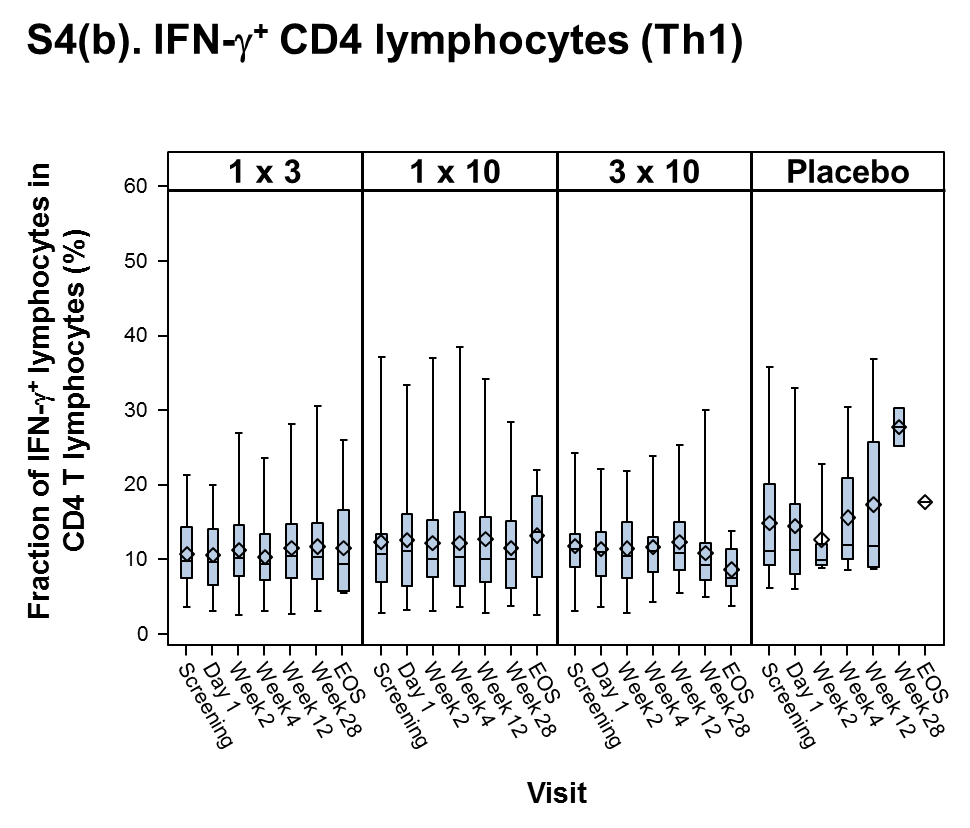
**

**
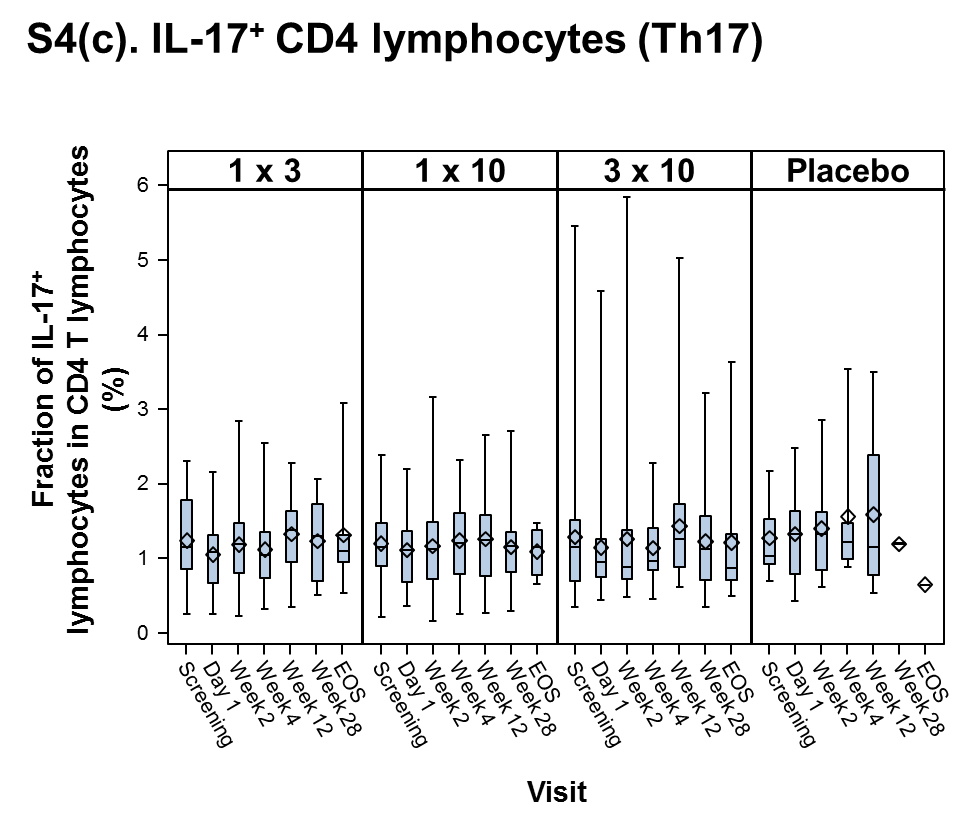
**

**
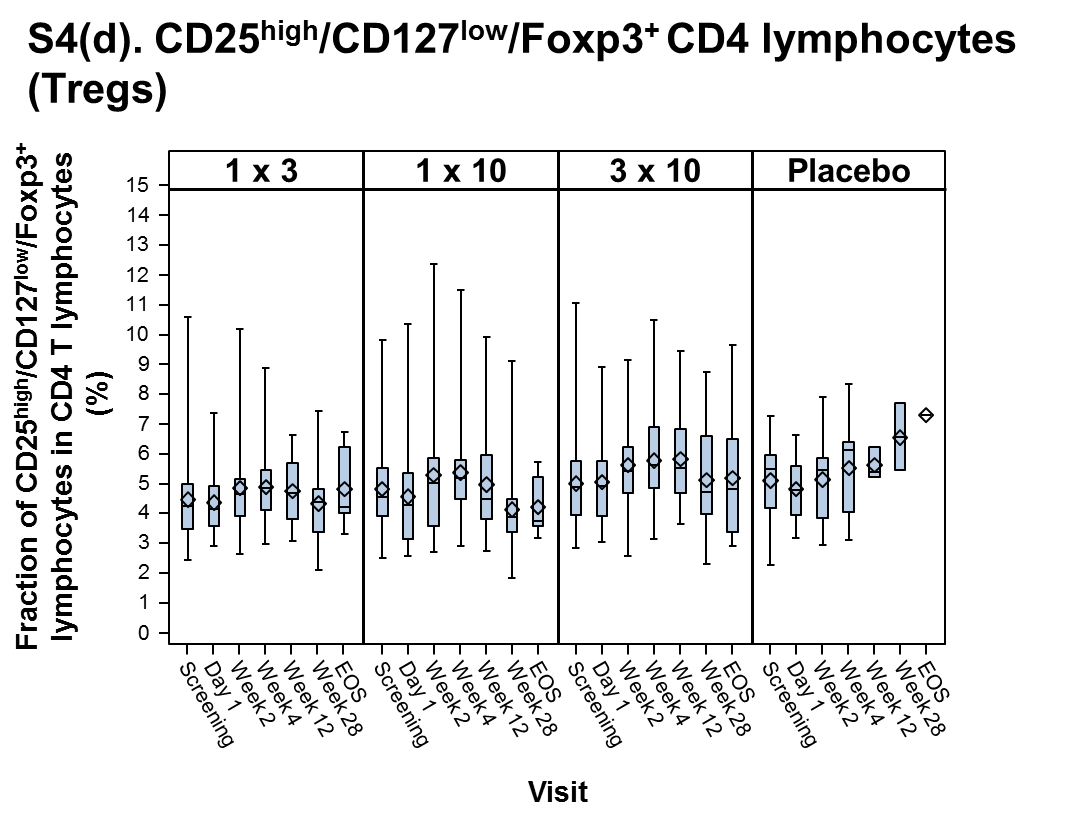
**

**Figure S5.** Examples of control stainings in immunohistochemistry and immunofluorescence. (a) Replacement of primary antibody (Ab) to interleukin (IL)-17 by goat immunoglobulin-G (AB-108-C, R&D Systems) and (b) of anti-myeloperoxidase (MPO) antibody by rabbit IgG (AB-105-C, R&D Systems) compared with active staining of the same area with anti–IL-17 polyclonal Ab (pAb) (c) and anti-MPO Ab (d). Panels (e) (anti–IL-17 staining) and (f) (anti-MPO staining) are enlarged sections of panels (c) and (d), respectively, depicting a Munro’s microabscess containing IL-17. In panel (i), anti–IL-17 pAb (5 μg) is pre-absorbed with 0.5 μg of recombinant human (rh)IL-17A (BTP21104; produced by Novartis Biologic Center), which completely abrogates IL-17 staining (shown in (g)) of mast cells detected by anti-tryptase Ab ((h); (j) corresponds to overlay of IL-17 and tryptase staining). The scale bar in (g) to (j) represents 15 μm. Blocking experiments were also done with serial dilutions of rhIL-17A and rhIL-17F and immunohistochemistry. In panel (m), anti–IL-17 pAb (2 μg/ml) was pre-absorbed with 2 μg/ml of rhIL-17A (R&D Systems), completely inhibiting IL-17 staining (shown in (k)) of epidermal neutrophils (open arrowheads) and dermal T cells and mast cells (closed arrowheads). Approximately ten times higher concentrations of rhIL-17F (R&D systems) were needed to obtain almost complete abrogation of staining with anti–IL-17 pAb (l). Panel (n) shows the isotype control for this set of experiments. Potential off-target binding of the goat anti–IL-17 pAb was also assessed more generally using a protein chip containing approximately 2960 human proteins spotted in duplicates (not shown). While binding to IL-17A, IL-17AF, and IL-1F was clearly detected, no off-target binding to other human proteins was detected by the protein chip analysis or subsequent confirmatory analysis of protein chip hits that had initially passed the cut-off criteria.

**S5(a-f).**


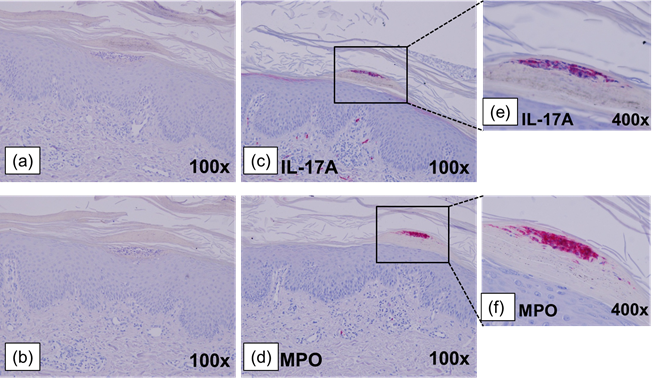


**S5(g-j).**

**
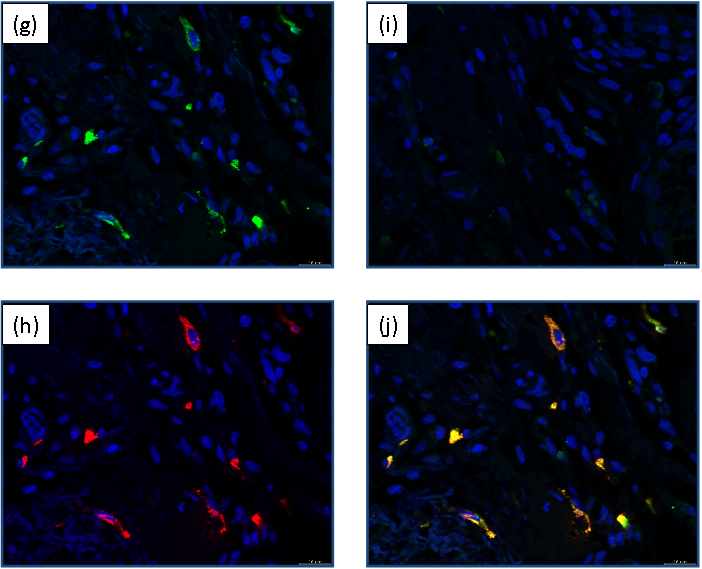
**

**S5(k-n)**

**
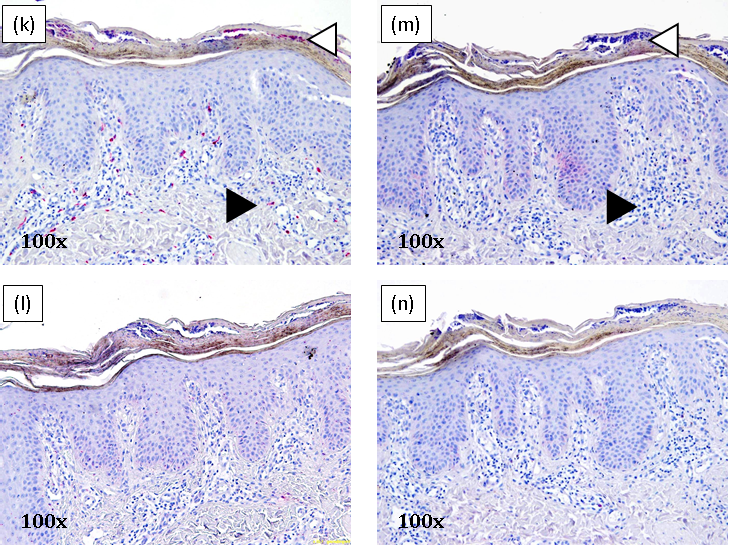
**

**Figure S6.** Assessment of additional epidermal and cellular markers over time. Changes are shown for (a) acanthosis, (b) Ki67, (c) mast cell tryptase and (d) epidermal and dermal myeloperoxidase (MPO) in subjects receiving secukinumab or placebo (PBO), as measured in biopsy specimens using a semi-quantitative scoring system. Solid lines indicate mean changes in Psoriasis Area and Severity Index (PASI); error bars are ± standard error of the mean.


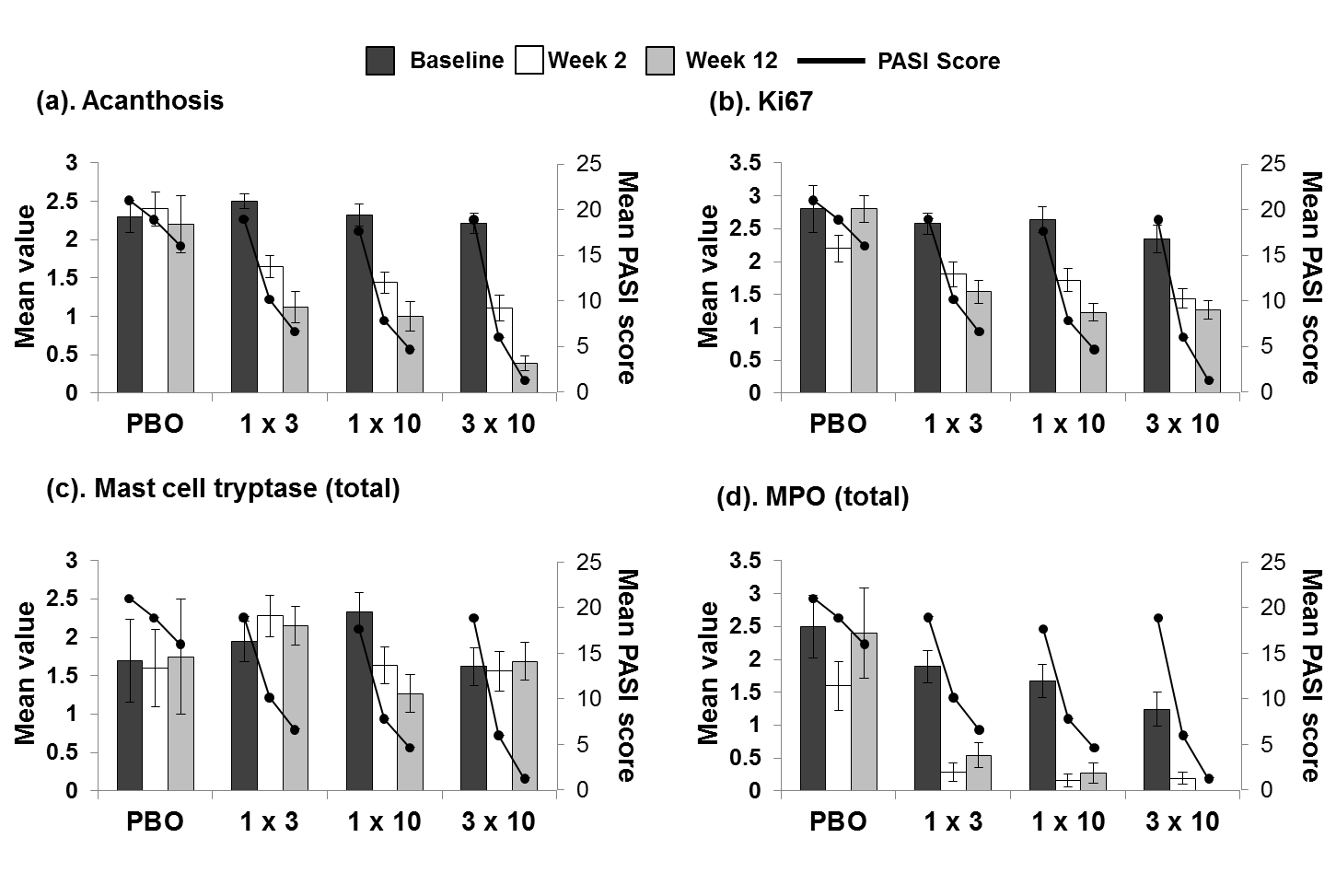


**Figure S7.** Proposed simplified model of psoriasis pathogenesis and early effects of anti–IL-17 therapy. (For details, see Discussion section of main paper.) GM-CSF, granulocyte-macrophage colony-stimulating factor; IL, interleukin; TNF, tumor necrosis factor.**
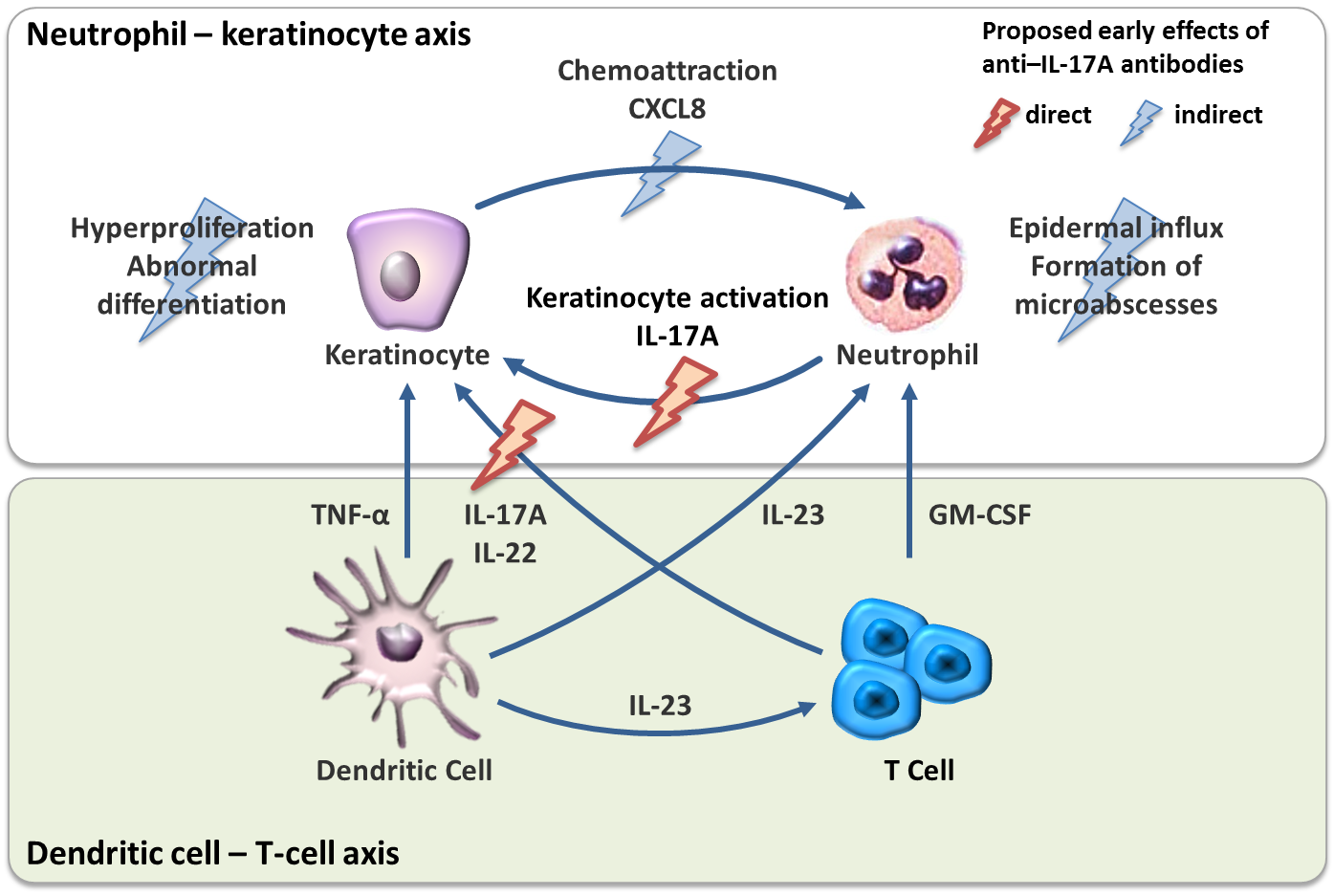
**

**Table S1.** NanoString nCounter Gene Expression Maestro CodeSet probe sequences for genes reported in this study

| **Gene** | **Capture probe sequence** | **Reporter probe sequence** |
| --- | --- | --- |
| *B2M* | CACGGAGCGAGACATCTCGGCCCGAATGCTGTCAGCTT | CAGGCCAGAAAGAGAGAGTAGCGCGAGCACAGCTAAGGC^1^ |
| *CXCL1* | CTCTATCACAGTGGCTGGCATGTTGCAGGCTCCTCAGAAATATTAACATA | TTCACAATGATCTCATTGGCCATTTGCTTGGATCCGCCAGC |
| *DSC2* | GGATCAACCGCAACAATCTCCGCAGATGACATGGTGGGTTTGCAGATGAT | TAGAACTCTCCAGACTAAAGTCAAAGGGTGGGCCATGGATAGGCTCATCA |
| *IFNG* | CTGGCTCAGATTGCAGGCATATTTTCAAACCGGCAGTAACTGGATAGTAT | ATCAGGGTCACCTGACACATTCAAGTTCTGTCTGACATGCCATTAAAGCA |
| *IL17A* | GGGTCCTCATTGCGGTGGAGATTCCAAGGTGAGGTG | GCACTTTGCCTCCCAGATCACAGAGGGATATCTCTCA |
| *IL17F* | ATTGATGATGCCAATGTCAAGCTTCATACTACCTCCTGGCACAGGCGGG | GAGGTGGAGCGGCTCTCGATGTTACGTGACATGGAAACGCGCTGGTTTTC |
| *IL8* | CCGGTGGTTTCTTCCTGGCTCTTGTCCTAGAAGCTTGTGTG | AGCCACGGCCAGCTTGGAAGTCATGTTTACACACAGTGAGATGGTTCCTT |
| *KRT1* | TTTGTTAGTATCTTGGTCCCTTAGATGAAGACTCGCCCACGGACC | ACCGTCGGGCGCCACCTCTTCAATAATTGTCTTGATTACTCTGGT^1^ |
| *KRT16* | GATGCTTGCTGGGAGGAAAGGTGGGCATCCTCGCCCTC | AAGAGGACGAGGAGGAGGTGAAGACCTCGCGGGAAGAATAGGATTGGCCA |
| *RPL13A* | TCCTTGCTCCCAGCTTCCTATGTCCCAGGGCTGCC | ATTCTCCGAGTGCTTTCAAGCAACTTCGGGAGGCAGTGACTAAGACCCTT |
| *RPL19* | AATCCTCATTCTCCTCATCCATGTGACCTTCTCTGGCATTCGGGCATTGG | TGGCGATCGATCTTCTTAGATTCACGGTATCTTCTGAGCAGCCGGCGCAA |
| *S100A7* | CATGTCGATCATGCCTATTATGGACCTCTCAGCTTGAGTGTTGCTCAT | AGCAGGCTTGGCTTGTCAATCTTGTCATCACGTCTGGTGTATTTGTGAAA^1^ |
| *S100A8* | AATTTCTTCAGGTCATCCCTGTAGACGGCATGGAAATTCCCCTTTATCAG | AGACGTCTGCACCCTTTTTCCTGATATACTGAGGACACTCGGTCTCTAGC^1^ |
| S100A9 | CCCAGCTTCACAGAGTATTGGTGGAAGGTGTTGATGATGGTCTCTATGTT | CTTTGAATTCCCCCTGGTTCAGGGTGTCTGGGTGC^1^ |
| *TNFA* | CAGGCCACACATTCCTGAATCCCAGGTTTCGAAGTGGTGGTCTTGTTGCT | TTCTGGAGGCCCCAGTTTGAATTCTTAGTGGTTGCCAGCACTTCACTGTG |
| *UBC* | CACTTCGAGAGTGATGGTCTTACCAGTCAGGGTCTTCACGAAGATCTGCA | TCCTTGTCTTGGATCTTTGCCTTGACATTCTCAATGGTGTCACTCGGCTC |

B2M, β2-microglobulin; DSC2, desmocollin-2; IFNG, interferon-γ; IL, interleukin; KRT, keratin; TNFA, tumor necrosis factor-α; UBC, ubiquitin C.

^1^Sequences of unlabeled standard desalted oligonucleotides used to compete with respective labeled reporter probes and prevent saturation of flow-cell imaging. (Probe sequences of all 191 targets contained in the Maestro CodeSet are available on request.)

**Table S2.** Demographic and Baseline clinical characteristics of study subjects

|  | **Secukinumab** | | | **Placebo** | **Total** |
| --- | --- | --- | --- | --- | --- |
|  | **1 x 3 mg/kg** | **1 x 10 mg/kg** | **3 x 10 mg/kg** |  |  |
| Safety analysis, *n*^1^ | 30 | 29 | 31 | 10 | 100 |
| Men, *n* (%) | 24 (80.0) | 22 (75.9) | 26 (83.9) | 8 (80.0) | 80 (80.0) |
| Mean age, y (SD) | 45.7 (9.3) | 43.5 (11.8) | 43.4 (10.9) | 40.1 (14.9) | 43.8 (11.1) |
| Mean BMI, kg/m^2^ (SD) | 31.3 (6.0) | 32.1 (8.3) | 31.0 (6.0) | 32.8 (14.4) | 31.6 (7.7) |
| Efficacy analysis, *n*^2^ | 30 | 25 | 29 | 10 | 94 |
| Mean PASI at Baseline (SD) | 19.0 (7.4) | 17.7 (5.1) | 18.9 (7.0) | 21.0 (9.5) | 18.8 (7.0) |
| Baseline IGA, *n* (%)  Mild  Moderate  Severe  Very severe | 0 (0)  18 (60)  11 (36.7) 1 (3.3) | 0 (0)  17 (68)  8 (32) 0 (0) | 1 (3.4)  16 (55.2)  12 (41.4) 0 (0) | 0 (0)  7 (70)  1 (10) 2 (20) | 1 (1)  58 (58)  32 (32)  3 (3) |

BMI, body mass index; IGA, Investigator’s Global Assessment; PASI, Psoriasis Area and Severity Index; SD, standard deviation.

^1^1 subject was wrongly allocated to dosing and reassigned for the safety analysis to another dosing cohort.

^2^Several subjects were removed from the efficacy analysis because of protocol deviations.
